# Supplementary material for: Quantitative assessments of retinal macular structure among rural-dwelling older adults in China: a population-based, cross-sectional, optical coherence tomography study
Source: BMJ Open. 2024 Feb 6;14(2):e079006. doi: 10.1136/bmjopen-2023-079006 (PMC10860037; doi:10.1136/bmjopen-2023-079006)
Supplement: Author's manuscript [file bmjopen-2023-079006.draft_revisions.pdf]

BMJ Open

BMJ Open is committed to open peer review. As part of this commitment we make the peer review history of every article we publish publicly available.

When an article is published we post the peer reviewers' comments and the authors' responses online. We also post the versions of the paper that were used during peer review. These are the versions that the peer review comments apply to.

The versions of the paper that follow are the versions that were submitted during the peer review process. They are not the versions of record or the final published versions. They should not be cited or distributed as the published version of this manuscript.

BMJ Open is an open access journal and the full, final, typeset and author-corrected version of record of the manuscript is available on our site with no access controls, subscription charges or pay-per-view fees (http://bmjopen.bmj.com).

If you have any questions on BMJ Open's open peer review process please email info.bmjopen@bmj.com

BMJ Open

Quantitative assessments of retinal macular structure among rural-dwelling Chinese older adults: a population-based optical coherence tomography study

Journal:	*BMJ Open*
Manuscript ID	bmjopen-2023-079006
Article Type:	Original research
Date Submitted by the Author:	22-Aug-2023
Complete List of Authors:	zhang, qinghua; Shandong Provincial Hospital<br>Zhang, Cong; Shandong Provincial Hospital affiliated to Shandong University, Department of Neurology<br>Wang, Yongxiang; Shandong Provincial Hospital affiliated to Shandong University, Department of Neurology; Shandong Provincial Hospital Affiliated to Shandong First Medical University, Department of Neurology<br>Cong, Lin; Shandong Provincial Hospital affiliated to Shandong University, Department of Neurology; Shandong Provincial Hospital Affiliated to Shandong First Medical University, Department of Neurology<br>Liu, Keke; Shandong Provincial Hospital affiliated to Shandong University, Department of Neurology; Shandong Provincial Hospital Affiliated to Shandong First Medical University, Department of Neurology<br>Xu, Zhe; Shandong Provincial Hospital affiliated to Shandong University, Department of Neurology<br>Jiang, Chunyan; Shandong Provincial Hospital Affiliated to Shandong First Medical University, Department of Neurology<br>Zhou, Weiyan; Shandong Provincial Hospital affiliated to Shandong University, Department of Ophthalmology; Shandong Provincial Hospital Affiliated to Shandong First Medical University, Department of Ophthalmology<br>Zhang, Chunxiao; Shandong Provincial Hospital affiliated to Shandong University, Department of Ophthalmology; Shandong Provincial Hospital Affiliated to Shandong First Medical University, Department of Ophthalmology<br>Dong, Yi; Shandong Provincial Hospital affiliated to Shandong University, Department of Neurology<br>Feng, Jianli; Shandong Provincial ENT Hospital, Department of Neurology<br>Qiu, Chengxuan; Shandong Provincial Hospital affiliated to Shandong University, Department of Neurology; Stockholm University, Department of Neurobiology, Care Sciences and Society<br>Du, YiFeng; Shandong Provincial Hospital affiliated to Shandong University, Department of Neurology; Shandong Provincial Hospital Affiliated to Shandong First Medical University, Department of Neurology
Keywords:	Epidemiology < TROPICAL MEDICINE, Aging, Neurology < INTERNAL MEDICINE, Diagnostic radiology < RADIOLOGY & IMAGING

SCHOLARONE™
Manuscripts

I, the Submitting Author has the right to grant and does grant on behalf of all authors of the Work (as defined in the below author licence), an exclusive licence and/or a non-exclusive licence for contributions from authors who are: i) UK Crown employees; ii) where BMJ has agreed a CC-BY licence shall apply, and/or iii) in accordance with the terms applicable for US Federal Government officers or employees acting as part of their official duties; on a worldwide, perpetual, irrevocable, royalty-free basis to BMJ Publishing Group Ltd ("BMJ") its licensees and where the relevant Journal is co-owned by BMJ to the co-owners of the Journal, to publish the Work in this journal and any other BMJ products and to exploit all rights, as set out in our licence.

The Submitting Author accepts and understands that any supply made under these terms is made by BMJ to the Submitting Author unless you are acting as an employee on behalf of your employer or a postgraduate student of an affiliated institution which is paying any applicable article publishing charge ("APC") for Open Access articles. Where the Submitting Author wishes to make the Work available on an Open Access basis (and intends to pay the relevant APC), the terms of reuse of such Open Access shall be governed by a Creative Commons licence – details of these licences and which Creative Commons licence will apply to this Work are set out in our licence referred to above.

Other than as permitted in any relevant BMJ Author's Self Archiving Policies, I confirm this Work has not been accepted for publication elsewhere, is not being considered for publication elsewhere and does not duplicate material already published. I confirm all authors consent to publication of this Work and authorise the granting of this licence.

For peer review only

Quantitative assessments of retinal macular structure among rural-dwelling Chinese older adults: a population-based optical coherence tomography study

Qinghua Zhang,[1,2] Cong Zhang,[1] Yongxiang Wang,[1,2] Lin Cong,[1,2] Keke Liu,[1,2] Zhe Xu,[1] Chunyan Jiang,[2] Weiyan Zhou,[3,4] Chunxiao Zhang,[3,4] Yi Dong,[1] Jianli Feng,[5] Chengxuan Qiu,[1,6*] Yifeng Du[1,2*]

1. Department of Neurology, Shandong Provincial Hospital affiliated to Shandong University, Jinan, Shandong, The People's Republic of China

2. Department of Neurology, Shandong Provincial Hospital affiliated to Shandong First Medical University, Jinan, Shandong, The People's Republic of China

3. Department of Ophthalmology, Shandong Provincial Hospital affiliated to Shandong University, Jinan, Shandong, The People's Republic of China

4. Department of Ophthalmology, Shandong Provincial Hospital affiliated to Shandong First Medical University, Jinan, Shandong, The People's Republic of China

5. Department of Neurology, Shandong Second Provincial General Hospital, Shandong Provincial ENT Hospital, Jinan, Shandong, The People's Republic of China

6. Aging Research Center and Center for Alzheimer Research, Department of Neurobiology, Care Sciences and Society, Karolinska Institutet-Stockholm University, Stockholm, Sweden

*Correspondence to: Yifeng Du, Department of Neurology, Shandong Provincial Hospital, Cheeloo College of Medicine, Shandong University, No. 324 Jingwuweiqi Road, Jinan, Shandong 250021, China. Email: duyifeng2013@163.com or Chengxuan Qiu, Email: chengxuan.qiu@ki.se.

For peer review only - http://bmjopen.bmj.com/site/about/guidelines.xhtml

Abstract

Background: To quantitatively assess and compare retinal macular structures of rural-dwelling older adults in China using two different optical coherence tomography (OCT) scanners and to examine their associations with demographic, lifestyle, clinical, and ocular factors.

Methods: This population-based cross-sectional study included 971 participants (age ≥60 years) derived from the multimodal interventions to delay dementia and disability in rural China (MIND-China). We collected data on demographics, lifestyle factors, clinical conditions (e.g., hypertension and diabetes), and ocular factors (e.g., visual acuity and intraocular pressure [IOP]). We used two kinds of spectral-domain OCT to measure macular parameters in nine ETDRS subfields. Data were analyzed using the multiple general linear models.

Results: Spectralis OCT demonstrated higher macular thickness but a lower macular volume than Primus 200 OCT (P<0.001). Nasal quadrant of the inner and outer subfields was the thickest, followed by superior quadrant. Adjusting for multiple potential confounding variables, older age was significantly correlated with lower average inner and outer macular thicknesses and overall macular volume (P<0.05). Men had higher macular parameters than women (P<0.05). Presence of cardiovascular disease (CVD) was correlated with lower central macular thickness (P<0.05). Middle school or above and lower spherical equivalent was correlated with higher average inner macular thickness (P<0.05).

Conclusions: Spectralis demonstrated with higher macular thickness but a lower macular volume. Older age and female sex are associated with lower macular thickness and volume. Macular parameters are associated with education, CVD, and spherical equivalent.

Keywords: **aging, retina, macular thickness, SD-OCT, population-based study**

For peer review only

For peer review only - http://bmjopen.bmj.com/site/about/guidelines.xhtml

What is already known on this topic- Differences in macular parameters by demographic factors (e.g., age and sex) are well known, but ocular and vascular risk factors in association with macular measurements in older adults have not yet been well studied. In addition, few population-based studies have assessed the differences and agreements of macular measurements across various models of SD-OCT scanners.

What this study adds- In this population-based study, we were able to directly compare the differences of macular parameters assessed using the two SD-OCT canners and the related factors. Our study targeted the largely ignored demographic groups of rural-dwelling older adults in China.

How this study might affect research, practice or policy- These data may help interpret macular parameters in the diagnosis and management of retinal disorders in older adults. More importantly, this study provides detailed description of baseline macular measurements in a subsample of the community-based MIND-China study, which will be highly relevant for future follow-up study of brain aging and functional phenotypes.

For peer review only - http://bmjopen.bmj.com/site/about/guidelines.xhtml

Background

The retina develops from diencephalon and is an extension of the central nervous system (CNS) that exhibits typical properties of the brain[1]. In patients with neurodegenerative disorders such as Parkinson disease (PD) and Alzheimer disease (AD), macular changes may precede onset of symptoms in the CNS diseases[2-4]. Thus, changes of macular structure may be useful markers for early detection of CNS disorders.

Optical coherence tomography (OCT) is a key non-invasive imaging technique that enables clinicians to quantitatively measure macular thickness and volume in different subfields. The imaging speed (40,000 axial scans per second) and image resolution (axial resolution up to 5 μm) of OCT are much higher than that of MRI scans (resolution <100-500 μm), while the expenditure is much lower[5]. Therefore, OCT scan may provide a comprehensive understanding of the macular structure that can be implemented in studies of the general population settings.

In 2010, the population-based Handan Eye Study used the time-domain OCT to describe normal macular parameters in rural Chinese adults (n=2230, age 30-85 years)[6]. However, OCT technology has advanced remarkably from time-domain OCT to spectral-domain OCT (SD-OCT), which may provide more precise segmentation and higher resolution and measurement of retina[7]. However, few population-based studies have assessed the differences and agreements of macular measurements across various models of SD-OCT scanners. In addition, differences in macular parameters by demographic factors (e.g., age and sex) are well known, but ocular and vascular risk factors in association with macular measurements in older adults have not yet been well studied[8-11].

In this population-based study among rural-dwelling older adults in China, we sought to describe the methodology of quantitative assessments of the macular parameters assessed with SD-OCT scanners, to compare macular parameters measured using two different SD-OCT scanners, and further to examine the relations of macular parameters with demographic features, ocular factors, and vascular risk factors.

Methods

For peer review only - http://bmjopen.bmj.com/site/about/guidelines.xhtml

Study population

The study sample was derived from participants in the baseline examination of the Multimodal Interventions to Delay Dementia and Disability in Rural China (MIND-China) study, as previously described in detail[12]. Briefly, MIND-China targeted local residents who were aged ≥60 years by the end of 2017 and living in the 52 villages of Yanlou Town, Yanggu County, western Shandong Province, China. Baseline multidisciplinary assessments were conducted in March-September 2018, during which 5765 participants were examined.

The SD-OCT substudy was embedded within MIND-China and conducted at two local hospitals from March to June 2018 in Yanlou Town Hospital using Primus 200 OCT scanner (Carl Zeiss Meditec, Germany) and from June 2019 to November 2020 in Southwest Lu Hospital using Spectralis OCT scanner (Software Version 1.10.2.0; Heidelberg Engineering, Heidelberg, Germany). Clustering-randomized sampling approach was used in the SD-OCT substudy. In total, 1083 subjects in Yanlou Town Hospital and 306 subjects in Southwest Lu Hospital underwent the SD-OCT scans. We excluded subjects due to the following reasons: OCT images with signal strength less than six for the Primus 200 and less than 25 for Spectralis; OCT images with centration and segmentation errors; subjects with self-reported history of neurological disease (e.g., multiple sclerosis); self-reported or evidence of glaucoma; retina or macular disease (e.g., diabetic retinopathy, age-related macular degeneration, epiretinal membrane and macular hole[13]), and history of ocular surgery and trauma. For OCT images acquired using Spectralis OCT scanner, right eye was preferred for final analysis. Finally, 700 subjects examined with the Primus 200 OCT, and 271 subjects with the Spectralis OCT were included in this analysis. **Figure 1** shows the flowchart of the study participants in the SD-OCT Substudy.

The protocol of the MIND-China study was reviewed and approved by the Ethics Committee at Shandong Provincial Hospital, Jinan, Shandong, China. Written informed consent was obtained from all participants, or in case of illiterate participants, from legally authorized representatives. MIND-China was registered in the Chinese Clinical Trial Registry (registration no.: ChiCTR1800017758, 13/08/2018).

For peer review only - http://bmjopen.bmj.com/site/about/guidelines.xhtml

[Fig. 1 about here]

Measurements and assessments of systemic factors

Data were collected via face-to-face interviews, clinical examination, laboratory test, and ocular examinations following the standard procedures[14]. Arterial blood pressure was measured using an electronic manometer (Omron HEM-7127J; Omron Corporation, Kyoto, Japan) after at least a 5-minute rest. After an overnight fast, peripheral blood samples were taken. Fasting serum glucose, total cholesterol, triglycerides, high-density lipoprotein cholesterol, low-density lipoprotein cholesterol were measured using an automatic Biochemical Analyzer (CS-600B, DIRUI Corporation, Changchun, China). Body mass index (BMI) was calculated as measured body weight (in kilograms) divided by body height (in meters) squared. We defined overweight as BMI 24-27.9 kg/m^2 and obesity as BMI ≥28 kg/m^2 according to recommendations for Chinese adults[15]. Smoking status was categorized into current, former, and never smoking. According to the frequency and quantity of alcohol consumption per week in the past 12 months, participants were divided into no or occasional drinkers, and regular drinkers (at least once a week). Regular alcohol drinkers were further divided into light-to-moderate drinkers (<21 standard drinks/week for men and <14 standard drinks/week for women) and heavy drinkers (≥21 standard drinks/week for men, ≥14 standard drinks/week for women)[16]. Physical inactivity was defined as fewer than 150 minutes per week spent in moderate or vigorous physical activities[14]. Hypertension was defined as systolic blood pressure ≥140 mmHg, or diastolic blood pressure ≥90 mmHg, or current use of antihypertensive drugs[17]. Diabetes was defined as having a fasting plasma blood glucose level ≥7.0 mmol/L, or using hypoglycemic drugs, or a self-reported history of diabetes[18]. Dyslipidemia was defined as having a high-density lipoprotein level <1.0 mmol/L, or low-density lipoprotein level ≥4.1 mmol/L, or triglycerides level ≥2.3 mmol/L, or total cholesterol ≥6.2 mmol/L, or using lipid-lowering drugs[14]. CVD was ascertained from in-person interviews, clinical and neurological examinations, and electrocardiogram examination, which included ischemic heart disease, heart failure, atrial fibrillation, and stroke[19].

Measurements of ocular factors

All participants underwent presenting visual acuity assessment and split-lamp examination. In

For peer review only - http://bmjopen.bmj.com/site/about/guidelines.xhtml

Southwest Lu Hospital, participants underwent additional eye examinations, including best corrected vision acuity, IOP, spherical equivalent, axial length and fundus imaging by trained ophthalmologist. Presenting and best corrected vision acuity were examined using standard logarithmic visual acuity chart (Chinese edition, GB11533-2011) at a 5-meter distance. Visual impairment was defined as presenting distance visual acuity <4.6 in the better eye[20]. Intraocular pressure was examined using non-contact tonometer (TX-20, Canon, Japan). Spherical equivalent was examined using computerized auto refractor (HDR-7000 system, Huvitz, Korea). Axial length was measured using A-scan ultrasound biometry (Compact TouchV.4.00, Quantel Medical, France). The anterior segment of the eye was examined using a handheld portable slit-lamp microscope (BM900, Haag-Streit Diagnostics, Switzerland). Both disc-centered and macular-centered fundus images were taken through undilated pupils using a hand-held direct ophthalmoscope (Suzhou Liuliu Vision Technology Co., Ltd., Suzhou, China).

Imaging of Optical Coherence Tomography

The same procedure for SD-OCT examination was followed in the two hospitals. Following ocular examination, the SD-OCT scan was performed by experienced ophthalmic technicians. (1) The Primus 200 OCT has an acquisition rate of 12000 A-scans per second, with an axial resolution of 5 μm in tissue and a transversal resolution of <20 μm using a super luminescence diode with an 870 nm bandwidth[21]. We scanned only one eye and the right eye was the first choice. The structure of macular retinal layers was analyzed following the macular thickness analysis protocol based on 512×32 macular cube scan in a dark room without pupil dilation, 6mm×6mm in scan area, centered at the fovea. (2) The Spectralis OCT has an acquisition rate of 40,000 A-scans per second, with an axial resolution of 5 μm in tissue and a transversal resolution of <6 μm using a super luminescence diode with an 870 nm bandwidth. The built-in TruTrack active eye-tacking technology, which compensates for eye movement artifact, will improve the reproducibility of foveal center selection during data acquisition[22]. Macular image was acquired using the perifoveal volumetric macular scan model (512 horizontal A scans per B scan, 49 horizontal B scans in 6 mm×6 mm area, no predetermined automatic real time) in a dark room without pupil dilation. The right eye was imaged first and then for the left eye.

For peer review only - http://bmjopen.bmj.com/site/about/guidelines.xhtml

Retinal thickness in the nine subfields defined by the Early Treatment Diabetic Retinopathy Study (ETDRS)[23] was acquired using the built-in software, as well as the general macular cube volume over the entire grid area. The standard retinal subfields are defined as central, inner superior, innerinferior, innernasal, innertemporal, outersuperior, outerinferior, outernasal, and outertemporal. The central foveal, inner, and outer subfields are bounded by the 1-mm, 3-mm, and 6-mm diameter circle, respectively. The whole retinal thickness was defined as the distance between internal limiting membrane (ILM) and retinal pigment epithelium boundary.

Evaluation of SD-OCT Images

Quality control measures included image quality score, an internal limiting membrane (ILM) indicator, and motion indicators, which helped identify cases with segmentation errors, blinks, and eye motion artifacts[21 24 25]. ILM indicator was a measure of the minimum localized edge strength around the ILM boundary across the entire scan. It was useful for identifying segmentation errors and blink artifacts. The image quality of SD-OCT scans was assessed based on the range of the quality scores assigned by the respective instrument. For Primus 200 OCT, signal strength varies from 0-10, signal strength of 6 or more is considered adequate for image analysis[21]. For Spectralis OCT, the quality factor is a measure of signal-to-noise ratio expressed on a scale of 1 to 40 decibels. A quality reading of 25 decibels or more is considered adequate for image analysis[22].

Statistical analysis

IBM SPSS Statistics for Windows, Version 25.0 (IBM Corp. Released 2017. Armonk, NY, USA) was used for all statistical analysis. We presented mean (standard deviation, SD) for continuous variables and frequency (%) for categorical variables. We used one-way analysis of variance (ANOVA) or t test to compare continuous variables, and chi-square test for categorical variables. Multiple general linear regression analyses were performed to examine the association of macular thickness and volumes (dependent variables) with various risk factors (independent variables). Adjustments were made first for age and sex in model 1, and additional adjustments were made for all other factors (i.e., education, smoking, alcohol consumption, CVD, and visual impairment) in model 2. The collinearity of the independent variables in model 2 was checked by calculating variance inflation factors (VIFs). Variables with high VIFs were excluded from the model. Data were missing in 3 participants (0.3%)

For peer review only - http://bmjopen.bmj.com/site/about/guidelines.xhtml

for hypertension, and 7 (0.7%) for physical activity. Missing values in categorical variables were replaced with a dummy variable. For all analyses, a 2-tailed P value of less than 0.05 was considered to be statistically significant.

Results

Characteristics of study participants

Characteristics of the participants by two types of SD-OCT scanners were summarized in **Table 1**. Compared with participants who were scanned with Primus 200 OCT, those scanned with Spectralis OCT were slightly younger (mean age 68.1 vs. 70.4 years; P<0.001) and less likely to have cardiovascular disease but more likely to have visual impairment (P<0.05). The two groups did not differ significantly in the distributions of sex, education, smoking, alcohol consumption, physical activity, BMI, hypertension, diabetes, and dyslipidemia.

[Table 1 about here]

Comparation of macular parameters assessed with Spectralis and Primus 200

Compared with macular parameters of participants examined with Primus 200 OCT scanner, those scanned with Spectralis OCT scanner had significantly higher macular thickness in all ETDRS subfields but a lower macular volume (P<0.001) (**Table 2**). The association of macular parameters with type of scanner remained significant even after adjusting for age, sex, and education (P<0.001).

[Table 2 about here]

Correlates of macular parameters

As shown in **Figure 2**, measurements of inner and outer macular thickness and macular volume were significantly decreased with increasing age in participants measured with both scanners (P<0.01). There was no significant difference with age in central macular thickness of both samples of participants scanned with Primus 200 OCT (P=0.643) and Spectralis OCT (P=0.695). In the multiple linear regression analysis, the association of age with macular thickness and volume remained

For peer review only - http://bmjopen.bmj.com/site/about/guidelines.xhtml

significant even after further adjusting for multiple potential confounding variables (P<0.01), but the association of age with average inner macular thickness became statistically non-significant after adjusting for multiple confounding variables in the sample of participants examined with Primus 200 OCT (**Table 3**).

Female sex was significantly associated with lower central, average inner and outer macular thickness, and macular volume after adjusting for age (P<0.05) in the sample of participants examined with both scanners, and the association of female sex with average inner and outer macular thickness and volume in the sample of participants examined with Spectralis OCT scanner and the association with central and average inner macular thickness in the sample of participants examined with Primus 200 OCT scanner remained significant even after further adjusting for multiple potential confounding variables (P<0.05) (**Table 3**).

In the sample of participants scanned with Spectralis OCT, middle school or above was significantly associated with higher central and average inner macular thickness after adjusting for sex and age (P<0.05), and the association with average inner macular thickness remained significant after further adjusting for multiple potential confounding variables (P<0.05) (**Table 3**). In addition, the presence of CVD was significantly associated with lower central macular thickness after adjusting for sex and age (P<0.05), but the association became statistically non-significant after further adjusting for multiple confounding factors. We also analyzed the associations of ocular factors (i.e., IOP, axial length, and spherical equivalent) with macular parameters in the subsample of participants examined with Spectralis OCT. Higher spherical equivalent was significantly associated with lower average inner macular thickness after adjusting for sex and age, but the association became statistically non-significant after further adjusting for multiple confounding factors (**Table 3**). No association was found between IOP, axial length and macular measurements, which was not shown in the table.

[Table 3 & Fig. 2 about here]

Discussion

In this community-based SD-OCT scan study, we quantified macular thickness and volume among rural-dwelling Chinese older adults (age ≥60 years) using two different SD-OCT scanners, compared

For peer review only - http://bmjopen.bmj.com/site/about/guidelines.xhtml

macular parameters examined with Spectralis and Primus 200 OCT, and further explored demographics, lifestyle factors, clinical conditions, and ocular factors associated with macular thickness and volume.

Macular parameters measured with different scanners vary substantially[26]. We found that, compared with Primus 200 OCT, Spectralis OCT scanner yielded higher macular thickness but a lower macular volume, which was consistent with the literature[26 27]. Different software algorithms were used in different OCT scanners, and the segmentation software of Spectralis OCT delineates the retinal pigment epithelium in a deeper layer than Primus 200 OCT [26]. This could be the main reason for higher macular thickness assessed with Spectralis OCT. In addition, the differences in macular parameters might be partially attributed to the fact that the sample of participants scanned with Spectralis OCT was younger than those scanned with Primus 200 OCT in our study.

We found that the macula was thinnest in the central subfields, followed by outer subfields and thickest in the inner subfields. Nasal quadrant of the inner and outer subfields was thickest, followed by the superior quadrant. These findings are in line with the distribution of neurons and synapse in the macular region of retina and consistent with reports from previous population-based studies [6 24 28-30]. The thicknesses of central, average inner and average outer macula of population scanned with Primus 200 OCT were slightly thinner than the reports from the Singapore Epidemiology of Eye Diseases Study of Chinese population (age ≥40 years)[28 29] and the UK Biobank study (age ≥40 years)[24]. However, macular parameters scanned with Spectralis OCT were thicker than that of the reports mentioned above. Differences in sociodemographic characteristics (e.g., age and sex) and OCT devices might partly contribute to the variation of macular thickness across studies[26].

In our study, older age was related to lower average inner and outer macular thickness and macular volume. There was a trend towards decreasing in central macular thickness with increasing age, but the association was not significant. These results were consistent with the reports from previous studies[24 28 29 31] and were also compatible with physiological characteristics of the retina[32 33]. Histological studies show that nerve fibers and ganglion cells with higher density in the peripheral part of the retina seem more vulnerable to loss with aging, while photoreceptors with higher density in the fovea are more resistant to age-related cell death[32 33].

For peer review only - http://bmjopen.bmj.com/site/about/guidelines.xhtml

Previous studies have consistently shown that women have thinner macular thickness in all subfields and smaller macular volume than men[6 24 28 30]. Our data showed similar sex differences in the examined macular parameters. Sex differences in macular thickness and volume may be attributed to hormonal changes at menopause, and this may explain why women were more likely than men to develop macular holes[30].

We also found that achieving middle school or above was associated with higher macular thickness in the sample of participants scanned with Spectralis OCT. Similarly, the UK Biobank study showed that higher education attainment was associated with higher thickness of macular retinal nerve fiber layer, ganglion cell complex, and ganglion cell-inner plexiform layer, but the association between overall macular thickness and education attainment was not reported in this study[34].

Several population-based studies have investigated the associations of lifestyle factors and clinical conditions with macular measurements[6 9 24 28-30], and found that alcohol consumption, smoking[24], high BMI[6 24], high fasting blood glucose[6] or diabetes[29], low levels of total cholesterol and triglyceride, and chronic kidney disease[29] were associated with lower macular measurements. Our data showed that having CVD was associated with lower central macular thickness in population scanned with Spectralis OCT. However, we did not find associations of common lifestyle factors and other clinical conditions with macular parameters. The exact reasons for the discrepant results are not clear, but the differences in sociodemographic features of study population and some methodological issues across studies might play a part. Of note, our study participants were derived from a typical rural area in China, where older people had low socioeconomic status, the majority of them received no or very limited education, and they had distinct lifestyles from those of urban populations. Pathophysiologically, macular thickness could be affected by hypertension[24 28 29]. The retina undergoes various pathophysiologic changes in patients with hypertension, such as vasoconstriction, vascular sclerosis, and exudative changes. Vasoconstriction and vascular sclerosis may contribute to macular thinning, while exudative changes may induce abnormal macular thickening[9 35]. Therefore, it is important to evaluate ocular fundus changes before OCT examination. However, in our study only participants examined with Spectralis OCT in Southwest Lu Hospital underwent fundus imaging evaluation. In addition, macular thickness is affected by diabetic retinopathy and its severity[29 36].

For peer review only - http://bmjopen.bmj.com/site/about/guidelines.xhtml

Unfortunately, diabetic retinopathy was not evaluated in our study. Further studies with careful assessment of diabetic retinopathy may help determine the relationships of macular measurements with diabetes and complications. Regular physical activity may promote ocular health, but no studies have analyzed the effect of physical activity on macular thickness. We found no association between physical inactivity and macular parameters among the rural-dwelling older adults.

Macular thickness and volume also varied with ocular factors. We found that lower spherical equivalent was associated with higher average inner macular thickness in the sample of participants scanned with Spectralis, which was consistent with other studies[24 37] and histologic findings of retinal thinning in myopic eyes[38 39].

The strengths of our study include the population-based design, the use of SD-OCT rather than TD–OCT scanners, and standardized assessment of wide range of systemic and ocular factors. Our study targeted older adults (age ≥60 years) who were living in the rural communities, whereas two previous studies of Chinese population investigated macular structures among middle-aged and elderly people from urban areas[6 29]. However, our study has limitations. First, ocular factors were acquired only in a small sample, thus, the study has limited power to analyze these factors. Second, the cross-sectional nature of our study does not allow us to make any causal inference for the observed associations between factors studied and macular parameters, and the observed cross-sectional associations might be subject to selective survival bias that usually leads to underestimation of the true associations. Third, we were not able to control for the potential confounding effects of some ocular diseases (e.g., age-related macular degeneration and diabetic retinopathy) due to lack of assessments of these disorders, and residual confounding might also exist due to imperfect assessments of some confounding factors.

For peer review only

Conclusion

In conclusion, this population-based SD-OCT study of older adults who were free of ocular disorders quantified the macular thicknesses and volumes. We found that older age and female sex were associated with thinner macular thickness. Macular parameters were also affected by education attainment, CVD， and spherical equivalent. Furthermore, Spectralis OCT demonstrated higher macular thickness but a lower macular volume than Primus 200 OCT. These data may help interpret

For peer review only - http://bmjopen.bmj.com/site/about/guidelines.xhtml

macular parameters in the diagnosis and management of retinal disorders in older adults. More importantly, this study provides detailed description of baseline macular measurements in a subsample of the community-based MIND-China study, which will be highly relevant for future follow-up study of brain aging and functional phenotypes.

List of abbreviations

SD, standard deviation; SD-OCT, spectral-domain optical coherence tomography; BMI, body mass index; MIND-China, multimodal interventions to delay dementia and disability in rural China; CVD, cardiovascular disease; IOP, intraocular pressure; ETDRS, early treatment diabetic retinopathy study; CNS, central nervous system; PD, Parkinson disease; AD, Alzheimer disease; MRI, magnetic resonance imaging; ILM, internal limiting membrane; SD, standard deviation; ANOVA, one-way analysis of variance.

Declarations

Ethics approval and consent to participate

The studies involving human participants were reviewed and approved by Ethics Committee at Shandong Provincial Hospital affiliated to Shandong University in Jinan, Shandong Province. All methods were performed in accordance with the relevant guidelines and regulations. All the patients/participants/legally authorized representatives of illiterate participants provided their written informed consent to participate in this study.

Consent for publication

Not applicable.

Availability of data and materials

The raw data supporting the conclusions of this article will be made available from the corresponding authors upon reasonable request and approval by the Steering Committee of MIND-China, without undue reservation.

For peer review only - http://bmjopen.bmj.com/site/about/guidelines.xhtml

372 **Competing interests**

373 The authors declare no financial or other conflicts of interest.

374 **Acknowledgements**

375 We would like to thank all participants of the MIND-China project as well as our research staff who were involved in the data collection and management.

377 **Funding**

378 This work was supported in part by grants from the National Key R&D Program of China (grant no.: 2017YFC1310100), the National Nature Science Foundation of China (grants no.: 82171175, 81861138008, and 81772448), the Academic Promotion Program of Shandong First Medical University, the Taishan Scholar Program of Shandong Province, Shandong Province postdoctoral innovation research program, and the Jinan Science and technology development plan (grant no. 201805076), China. C Qiu received grants from the Swedish Research Council (VR, grants no.: 2017-05819 and 2020-01574), and the Swedish Foundation for International Cooperation in Research and Higher Education (STINT, grant no.: CH2019-8320), and Karolinska Institutet, Stockholm, Sweden. The funding agency had no role in the study design, data collection and analysis, the writing of this manuscript, and in the decision to submit the work for publication.

388 **Authors' contributions**

389 QZ, LC, YW, YDu, and CQ contributed to the study concept and design. QZ, CZ, YW, ZX, CJ, YZ, CZ, YDong, and JF contributed to the acquisition and assessment of data. QZ and KL contributed to the data analysis. QZ contributed to the drafting of the manuscript. YDu and CQ contributed to the supervision. All authors contributed to the critical revision of the manuscript for important intellectual content, article, and approved the submitted version.

For peer review only - http://bmjopen.bmj.com/site/about/guidelines.xhtml

References

1. Cheung CY-L, Ikram MK, Chen C, et al. Imaging retina to study dementia and stroke. *Prog Retin Eye Res* 2017;57.
2. Ito Y, Sasaki M, Takahashi H, et al. Quantitative Assessment of the Retina Using OCT and Associations with Cognitive Function. *Ophthalmology* 2020;127(1):107-18.
3. London A, Benhar I, Schwartz M. The retina as a window to the brain-from eye research to CNS disorders. *Nat Rev Neurol* 2013;9(1):44-53.
4. Mutlu U, Colijn JM, Ikram MA, et al. Association of Retinal Neurodegeneration on Optical Coherence Tomography With Dementia: A Population-Based Study. *JAMA Neurol* 2018;75(10):1256-63.
5. Cabrera DeBuc D, Gaca-Wysocka M, Grzybowski A, et al. Identification of Retinal Biomarkers in Alzheimer's Disease Using Optical Coherence Tomography: Recent Insights, Challenges, and Opportunities. *J Clin Med* 2019;8(7) .
6. Duan XR, Liang YB, Friedman DS, et al. Normal macular thickness measurements using optical coherence tomography in healthy eyes of adult Chinese persons: the Handan Eye Study. *Ophthalmology* 2010;117(8):1585-94.
7. Seibold LK, Mandava N, Kahook MY. Comparison of retinal nerve fiber layer thickness in normal eyes using time-domain and spectral-domain optical coherence tomography. *Am J Ophthalmol* 2010;150(6):807-14.
8. Ooto S, Hangai M, Tomidokoro A, et al. Effects of age, sex, and axial length on the three-dimensional profile of normal macular layer structures. *Invest Ophthalmol Vis Sci* 2011;52(12):8769-79.
9. Kong M, Kwun Y, Sung J, et al. Association Between Systemic Hypertension and Macular Thickness Measured by Optical Coherence Tomography. *Invest Ophthalmol Vis Sci* 2015;56(4):2144-50.
10. Sasaki M, Kawashima M, Kawasaki R, et al. Association of serum lipids with macular thickness and volume in type 2 diabetes without diabetic macular edema. *Invest Ophthalmol Vis Sci* 2014;55(3):1749-53.
11. Sng CCA, Cheung CY, Man RE, et al. Influence of diabetes on macular thickness measured using optical coherence tomography: the Singapore Indian Eye Study. *Eye (Lond)* 2012;26(5):690-98.
12. Wang Y, Han X, Zhang X, et al. Health status and risk profiles for brain aging of rural-dwelling older adults: Data from the interdisciplinary baseline assessments in MIND-China. *Alzheimers Dement (N Y)* 2022;8(1):e12254.
13. Müller PL, Wolf S, Dolz-Marco R, et al. Ophthalmic Diagnostic Imaging: Retina. In: Bille JF, ed. High Resolution Imaging in Microscopy and Ophthalmology: New Frontiers in Biomedical Optics. Cham CH: 2019, The Author(s). 2019:87-106.
14. Han X, Jiang Z, Li Y, et al. Sex disparities in cardiovascular health metrics among rural-dwelling older adults in China: a population-based study. *BMC Geriatr* 2021;21(1):158.
15. Zhou B-F. Predictive values of body mass index and waist circumference for risk factors of certain related diseases in Chinese adults--study on optimal cut-off points of body mass index and waist circumference in Chinese adults. *Biomed Environ Sci* 2002;15(1):83-96.
16. Järvenpää T, Rinne JO, Koskenvuo M, et al. Binge drinking in midlife and dementia risk. *Epidemiology* 2005;16(6):766-71.
17. Jia P, Lee H, Chan J, et al. Long-Term Blood Pressure Variability Increases Risks of Dementia and Cognitive Decline: A Meta-Analysis of Longitudinal Studies. *Hypertension (Dallas, Tex : 1979)* 2021:HYPERTENSIONAHA12117788. doi: 10.1161/hypertensionaha.121.17788

For peer review only

For peer review only - http://bmjopen.bmj.com/site/about/guidelines.xhtml

18. Liu X, Li Y, Li L, et al. Prevalence, awareness, treatment, control of type 2 diabetes mellitus and risk factors in Chinese rural population: the RuralDiab study. *Sci Rep* 2016;6:31426.
19. Cong L, Ren Y, Hou T, et al. Use of Cardiovascular Drugs for Primary and Secondary Prevention of Cardiovascular Disease Among Rural-Dwelling Older Chinese Adults. *Front Pharmacol* 2020;11:608136.
20. Vitale S, Cotch MF, Sperduto RD. Prevalence of visual impairment in the United States. *JAMA* 2006;295(18):2158-63.
21. Balasubramanian M, Bowd C, Vizzeri G, et al. Effect of image quality on tissue thickness measurements obtained with spectral domain-optical coherence tomography. *Opt Express* 2009;17(5):4019-36.
22. Ctori I, Huntjens B. Repeatability of Foveal Measurements Using Spectralis Optical Coherence Tomography Segmentation Software. *PLoS One* 2015;10(6):e0129005.
23. Relhan N, Flynn HW. The Early Treatment Diabetic Retinopathy Study historical review and relevance to today's management of diabetic macular edema. *Curr Opin Ophthalmol* 2017;28(3):205-12.
24. Patel PJ, Foster PJ, Grossi CM, et al. Spectral-Domain Optical Coherence Tomography Imaging in 67 321 Adults: Associations with Macular Thickness in the UK Biobank Study. *Ophthalmology* 2016;123(4):829-40.
25. Tewarie P, Balk L, Costello F, et al. The OSCAR-IB consensus criteria for retinal OCT quality assessment. *PLoS One* 2012;7(4):e34823.
26. Mylonas G, Ahlers C, Malamos P, et al. Comparison of retinal thickness measurements and segmentation performance of four different spectral and time domain OCT devices in neovascular age-related macular degeneration. *Br J Ophthalmol* 2009;93(11):1453-60.
27. Miller M, Zemon V, Nolan-Kenney R, et al. Optical coherence tomography of the retina in schizophrenia: Inter-device agreement and relations with perceptual function. *Schizophr Res* 2020;219:13-18.
28. Gupta P, Sidhartha E, Tham YC, et al. Determinants of macular thickness using spectral domain optical coherence tomography in healthy eyes: the Singapore Chinese Eye study. *Invest Ophthalmol Vis Sci* 2013;54(13):7968-76.
29. Wong KH, Tham Y-C, Nguyen DQ, et al. Racial differences and determinants of macular thickness profiles in multiethnic Asian population: the Singapore Epidemiology of Eye Diseases Study. *Br J Ophthalmol* 2019;103(7):894-99.
30. Barrio-Barrio J, Ruiz-Canela M, Noval S, et al. Retinal Thickness Measured by Spectral-Domain Optical Coherence Tomography in Eyes Without Retinal Abnormalities: The Beaver Dam Eye Study. *Am J Ophthalmol* 2015;160(1):209-10.
31. Subhi Y, Forshaw T, Sørensen TL. Macular thickness and volume in the elderly: A systematic review. *Ageing Res Rev* 2016;29:42-49. doi: 10.1016/j.arr.2016.05.013
32. Gao H, Hollyfield JG. Aging of the human retina. Differential loss of neurons and retinal pigment epithelial cells. *Invest Ophthalmol Vis Sci* 1992;33(1)
33. Panda-Jonas S, Jonas JB, Jakobczyk-Zmija M. Retinal photoreceptor density decreases with age. *Ophthalmology* 1995;102(12):1853-59.
34. Khawaja AP, Chua S, Hysi PG, et al. Comparison of Associations with Different Macular Inner Retinal Thickness Parameters in a Large Cohort: The UK Biobank. *Ophthalmology* 2020;127(1):62-71.
35. Ahn SJ, Woo SJ, Park KH. Retinal and choroidal changes with severe hypertension and their association with visual outcome. *Invest Ophthalmol Vis Sci* 2014;55(12):7775-85.
36. Dai W, Tham YC, Cheung N, et al. Macular thickness profile and diabetic retinopathy: the Singapore

For peer review only - http://bmjopen.bmj.com/site/about/guidelines.xhtml

Epidemiology of Eye Diseases Study. *Br J Ophthalmol* 2018;102(8):1072-76.
37. von Hanno T, Lade AC, Mathiesen EB, et al. Macular thickness in healthy eyes of adults (N = 4508) and relation to sex, age and refraction: the Tromsø Eye Study (2007-2008). *Acta Ophthalmol* 2017;95(3):262-69.
38. Chui TYP, Song H, Burns SA. Individual variations in human cone photoreceptor packing density: variations with refractive error. *Invest Ophthalmol Vis Sci* 2008;49(10):4679-87.
39. Grosvenor T, Scott R. Three-year changes in refraction and its components in youth-onset and early adult-onset myopia. *Optom Vis Sci* 1993;70(8):677-83.

For peer review only

For peer review only - http://bmjopen.bmj.com/site/about/guidelines.xhtml

Figure 1. Flowchart of study participants in the MIND-China SD-OCT substudy

Abbreviations: MIND-China, the Multimodal Interventions to Delay Dementia and Disability in Rural China; SD-OCT, spectral-domain optical coherence tomography.

Figure 2. Distributions of macular thicknesses and volume by age groups assessed with Spectralis and Primus 200 OCT scanners. Data are mean (standard deviation). (A) Central macular thickness, (B) average inner macular thickness, (C) average outer macular thickness, and (D) macular volume.

For peer review only

For peer review only - http://bmjopen.bmj.com/site/about/guidelines.xhtml

518 **Table 1.** Characteristics of the study participants in the analytical samples by SD-OCT scanners

Characteristics	Primus 200 (n=700)	Spectralis (n=271)	P-value
**Demographic factors**			
Age, y, mean (SD)	70.4(4.2)	68.1(4.1)	<0.001
Female sex, n (%)	381(54.4)	153(56.5)	0.569
Education			0.114
Illiterate	285(40.7)	91(33.6)	
Elementary school	297(42.4)	126(46.5)	
Middle school or above	118(16.9)	54(19.9)	
**Lifestyle factors**			
Smoking status, n (%)			0.110
Never	451(64.4)	192(70.8)	
Former	100(14.3)	27(10.0)	
Current	149(21.3)	52(19.2)	
Alcohol consumption, n (%)			0.657
No or occasional	495(70.7)	188(69.4)	
Light-to-moderate	169(24.1)	65(24.0)	
Heavy	36(5.1)	18(6.6)	
Physical inactivity, n (%)	447(63.9)	181(66.8)	0.391
**Clinical conditions**			
BMI (kg/m^2), n (%)			0.684
Normal (<24)	268(38.3)	112(41.3)	
Overweight (24-27.9)	291(41.6)	107(39.5)	
Obesity (≥28)	141(20.1)	52(19.2)	
Hypertension, n (%)	475(67.9)	186(68.8)	0.816
Diabetes, n (%)	94(13.4)	31(11.4)	0.406
Dyslipidemia, n (%)	177(25.3)	60(22.1)	0.306
Cardiovascular disease, n (%)	265(37.9)	68(25.1)	<0.001
Visual impairment	28(4.0)	21(7.7)	0.017

519 Abbreviations: SD, standard deviation; SD-OCT, spectral-domain optical coherence tomography;
520 BMI, body mass index.
521 In the analytical sample, data were missing in 3 participants (0.3%) for hypertension and 7 (0.7%) for
522 physical activity. Missing values in categorical variables were replaced with a dummy variable.
523 P-value is for the test of differences between people scanned with Spectralis OCT and those scanned
524 with Primus 200 OCT.
525
526
527

For peer review only - http://bmjopen.bmj.com/site/about/guidelines.xhtml

Table 2. Mean (SD) of macular thickness and volume in different macular subfields measured with Spectralis and Primus 200 OCT scanners.

Macular parameters	Primus 200[a] (n=700)	Spectralis[a] (n=271)	β coefficient (95% CI)[b]	P-value
Macular thickness(μm)				
Central macular subfield	245.6(26.0)	263.7(22.9)	17.98(14.43,21.53)	<0.001
Inner macular subfields				
Superior	310.9(23.3)	331.7(18.0)	19.55(16.43,22.67)	<0.001
Inferior	308.8(25.6)	327.6(20.8)	18.08(14.60,21.55)	<0.001
Nasal	314.8(20.2)	334.3(19.4)	18.86(16.07,21.66)	<0.001
Temporal	303.5(19.5)	319.9(18.5)	15.58(12.88,18.29)	<0.001
Average inner macular	309.5(18.6)	328.4(18.3)	18.02(15.42,20.61)	<0.001
Outer macular subfields				
Superior	277.6(20.9)	293.1(15.3)	13.79(10.99,16.59)	<0.001
Inferior	258.8(23.9)	283.9(15.7)	24.15(20.99,27.32)	<0.001
Nasal	289.2(19.3)	310.6(16.4)	20.13(17.48,22.79)	<0.001
Temporal	260.0(16.4)	281.0(14.6)	19.81(17.54,22.08)	<0.001
Average outer macular	271.4(15.3)	292.2(14.0)	19.47(17.35,21.60)	<0.001
Macular volume(mm^3)	9.8(0.6)	8.5(0.4)	-1.34(-1.42, -1.26)	<0.001

Abbreviation: OCT, optical coherence tomography.

[a]Data are mean (standard deviation).

[b]β coefficient (95% confidence interval) of macular parameters associated with scanner (Spectralis vs. Primus 200), controlling for age, sex, and education.

For peer review only

For peer review only - http://bmjopen.bmj.com/site/about/guidelines.xhtml

Table 3. β coefficients (95% confidence intervals) of macular measurements associated with systemic and ocular factors from different models

Factors	Central macular (μm)		Average inner macular (μm)		Average outer macular (μm)		Volume (mm^3)	
	Model 1[a]	Model 2[a]	Model 1[a]	Model 2[a]	Model 1[a]	Model 2[a]	Model 1[a]	Model 2[a]
**Spectralis OCT (n=271)**								
Age (per 5 years)	-1.46 (-4.78, 1.85)	-1.01 (-4.36,2.33)	-3.62 (-6.17,-1.06)*	-3.34 (-5.91,-0.77)*	-3.43 (-5.42,-1.44)*	-3.39 (-5.42,-1.36)*	-0.10 (-0.15,-0.04)*	-0.10 (-0.15,-0.04)*
Sex (female vs. male)	-10.45 (-15.86,-5.03)*	-5.32 (-13.67,3.03)	-11.63 (-15.80,-7.45)*	-10.20 (-16.61,-3.80)*	-5.89 (-9.15,-2.63)*	-5.13 (-10.19,-0.06)*	-0.21 (-0.30,-0.11)*	-0.18 (-0.32,-0.03)*
Education								
Illiterate	0.00 (ref.)	0.00 (ref.)	0.00 (ref.)	0.00 (ref.)	0.00 (ref.)	0.00 (ref.)	0.00 (ref.)	0.00 (ref.)
Elementary school	3.10 (-3.22,9.42)	2.96 (-3.44,9.35)	3.70 (-1.16, 8.55)	3.81 (-1.09,8.72)	2.12 (-1.70, 5.94)	2.35 (-1.52,6.23)	0.07 (-0.04, 0.18)	0.08 (-0.03,0.19)
Middle school or above	8.86 (0.27,17.44)*	8.24 (-0.50,16.98)	8.49 (1.90,15.09)*	7.85 (1.14,14.55)*	3.76 (-1.43, 8.94)	3.61 (-1.69,8.91)	0.14 (-0.01, 0.28)	0.13 (-0.02,0.28)
Smoking								
Never	0.00 (ref.)	0.00 (ref.)	0.00 (ref.)	0.00 (ref.)	0.00 (ref.)	0.00 (ref.)	0.00 (ref.)	0.00 (ref.)
Former	-2.84 (-13.57,7.89)	-3.90 (-14.85,7.05)	-3.21 (-11.52,5.10)	-4.32 (-12.72,4.08)	0.08 (-6.41, 6.58)	0.14 (-6.50,6.78)	-0.02 (-0.20, 0.17)	-0.02 (-0.21,0.16)
Current	4.93 (-3.78,13.65)	3.37 (-5.76,12.50)	-0.16 (-6.91, 6.58)	-1.31 (-8.32,5.69)	0.71 (-4.56, 5.98)	0.58 (-4.96,6.12)	0.02 (-0.13, 0.17)	0.01 (-0.15,0.16)
Alcohol consumption								
No or occasional	0.00 (ref.)	0.00 (ref.)	0.00 (ref.)	0.00 (ref.)	0.00 (ref.)	0.00 (ref.)	0.00 (ref.)	0.00 (ref.)
Light-to-moderate	3.39 (-4.29,11.06)	1.74 (-6.17,9.65)	0.36 (-5.57, 6.29)	-0.69 (-6.77,5.38)	-1.73 (-6.35, 2.89)	-2.53 (-7.33,2.27)	-0.03 (-0.16, 0.10)	-0.06 (-0.19,0.08)
Heavy	3.94 (-7.83,15.71)	1.68 (-10.50,13.86)	0.64 (-8.45, 9.74)	0.81 (-8.54,10.16)	0.64 (-6.45, 7.72)	0.58 (-6.81,7.97)	0.02 (-0.18, 0.22)	0.02 (-0.19,0.23)
Cardiovascular disease	-6.83 (-13.08,-0.58)*	-6.05 (-12.41,0.31)	-3.09 (-7.94, 1.76)	-2.59 (-7.47,2.29)	-1.09 (-4.88, 2.70)	-1.01 (-4.87,2.85)	-0.05 (-0.16, 0.06)	-0.04 (-0.15,0.07)

For peer review only

For peer review only - http://bmjopen.bmj.com/site/about/guidelines.xhtml

Vision impairment	-1.50 (-11.61,8.62）	-0.03 (-10.26,10.20)	-4.79 (-12.58,2.99)	-4.67 (-12.51,3.18)	-1.13 (-7.22, 4.96)	-1.07 (-7.28,5.14)	-0.05 (-0.23, 0.12)	-0.05 (-0.23,0.13)
Spherical equivalent	-0.27 (-2.51,1.97）	-0.09 (-2.34,2.16)	-1.78 (-3.50,-0.07)*	-1.67 (-3.39,0.06)	-1.17 (-2.51, 0.17)	-1.15 (-2.51,0.21)	-0.04 (-0.07, 0.00)	-0.04 (-0.07,0.00)
**Primus 200 OCT (n=700)**								
Age (per 5 years)	-0.08 (-2.31,2.15)	0.10 (-2.16,2.36)	-1.47 (-3.07,0.12)	-1.36 (-2.98,0.26)	-2.34 (-3.67,-1.01)*	-2.32 (-3.67,-0.97)*	-0.07 (-0.12,-0.02)*	-0.06 (-0.12,-0.01)*
Sex (female vs. male)	-13.31 (-17.07,-9.55)*	-11.06 (-18.10,-4.02)*	-8.72 (-11.41,-6.02)*	-7.47 (-12.51,-2.43)*	-4.32 (-6.57,-2.08)*	-3.33 (-7.54,0.88)	-0.15 (-0.24,-0.07)*	-0.12 (-0.28,0.04)
Education								
Illiterate	0.00 (ref.)	0.00 (ref.)	0.00 (ref.)	0.00 (ref.)	0.00 (ref.)	0.00 (ref.)	0.00 (ref.)	0.00 (ref.)
Elementary school	-1.90 (-6.39,2.59)	-2.02 (-6.55,2.50)	-0.35 (-3.57,2.88)	-0.58 (-3.82,2.66)	0.43 (-2.25, 3.12)	0.34 (-2.37,3.04)	0.00 (-0.10, 0.10)	-0.01 (-0.11,0.10)
Middle school or above	2.93 (-3.32,9.17)	2.94 (-3.34,9.22)	0.88 (-3.59,5.36)	0.62 (-3.87,5.12)	2.05 (-1.68, 5.78)	1.92 (-1.83,5.67)	0.09 (-0.05, 0.23)	0.08 (-0.06,0.22)
Smoking								
Never	0.00 (ref.)	0.00 (ref.)	0.00 (ref.)	0.00 (ref.)	0.00 (ref.)	0.00 (ref.)	0.00 (ref.)	0.00 (ref.)
Former	1.50 (-6.16,9.15)	2.24 (-5.49,9.98)	2.90 (-2.58,8.37)	2.93 (-2.61,8.47)	1.58 (-2.99, 6.14)	1.83 (-2.79,6.46)	0.06 (-0.11, 0.24)	0.07 (-0.11,0.24)
Current	3.31 (-3.79,10.40)	4.32 (-2.97,11.60)	-0.31 (-5.38,4.76)	-0.11 (-5.33,5.11)	-1.22 (-5.44, 3.01)	-1.14 (-5.50,3.21)	-0.08 (-0.24, 0.08)	-0.08 (-0.24,0.09)
Alcohol consumption								
No or occasional	0.00 (ref.)	0.00 (ref.)	0.00 (ref.)	0.00 (ref.)	0.00 (ref.)	0.00 (ref.)	0.00 (ref.)	0.00 (ref.)
Light-to-moderate	-2.34 (-7.52,2.85)	-2.71 (-8.00,2.57)	0.55 (-3.17,4.26)	0.64 (-3.15,4.43)	0.53 (-2.57, 3.63)	0.66 (-2.50,3.82)	0.03 (-0.09, 0.15)	0.04 (-0.08,0.16)
Heavy	1.52 (-7.65,10.68)	0.59 (-8.74,9.93)	-1.41 (-7.97,5.15)	-1.12 (-7.81,5.56)	-0.28 (-5.75, 5.19)	0.03 (-5.55,5.61)	0.01 (-0.20,0.21)	0.03 (-0.18,0.24)
Cardiovascular disease	-1.40 (-5.27,2.47)	-1.51 (-5.41,2.39)	-0.35 (-3.12,2.42)	-0.52 (-3.31,2.27)	-1.27 (-3.58, 1.03)	-1.39 (-3.72,0.94)	-0.03 (-0.12,0.06)	-0.03 (-0.12,0.05)
Vision impairment	-4.47 (-14.09,5.15)	-4.67 (-14.32,4.99)	-6.39 (-13.26, 0.48)	-6.19 (-13.10,0.73)	-0.54 (-6.28, 5.21)	-0.17 (-5.94,5.61)	-0.14 (-0.35, 0.08)	-0.12 (-0.34,0.10)

For peer review only

For peer review only - http://bmjopen.bmj.com/site/about/guidelines.xhtml

535 [a]β coefficients (95% confidence intervals) were adjusted for age and sex in model 1; and additionally adjusted for all other factors in the table in model 2.

536 *$P<0.05$.

For peer review only

For peer review only - http://bmjopen.bmj.com/site/about/guidelines.xhtml

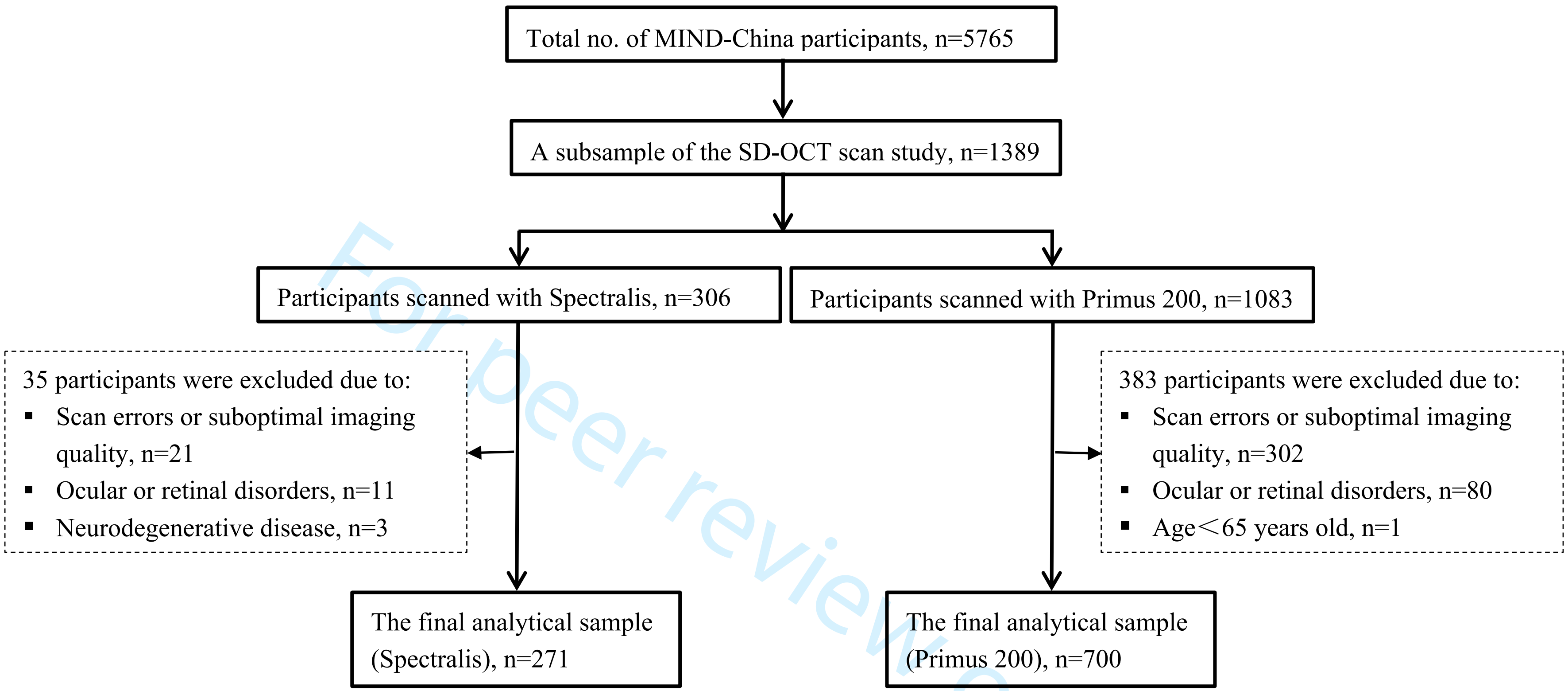

Figure 1. Flowchart of study participants in the MIND-China SD-OCT substudy

Abbreviations: MIND-China, multimodal interventions to delay dementia and disability in rural China; SD-OCT, spectral-domain optical coherence tomography.

For peer review only

For peer review only - http://bmjopen.bmj.com/site/about/guidelines.xhtml

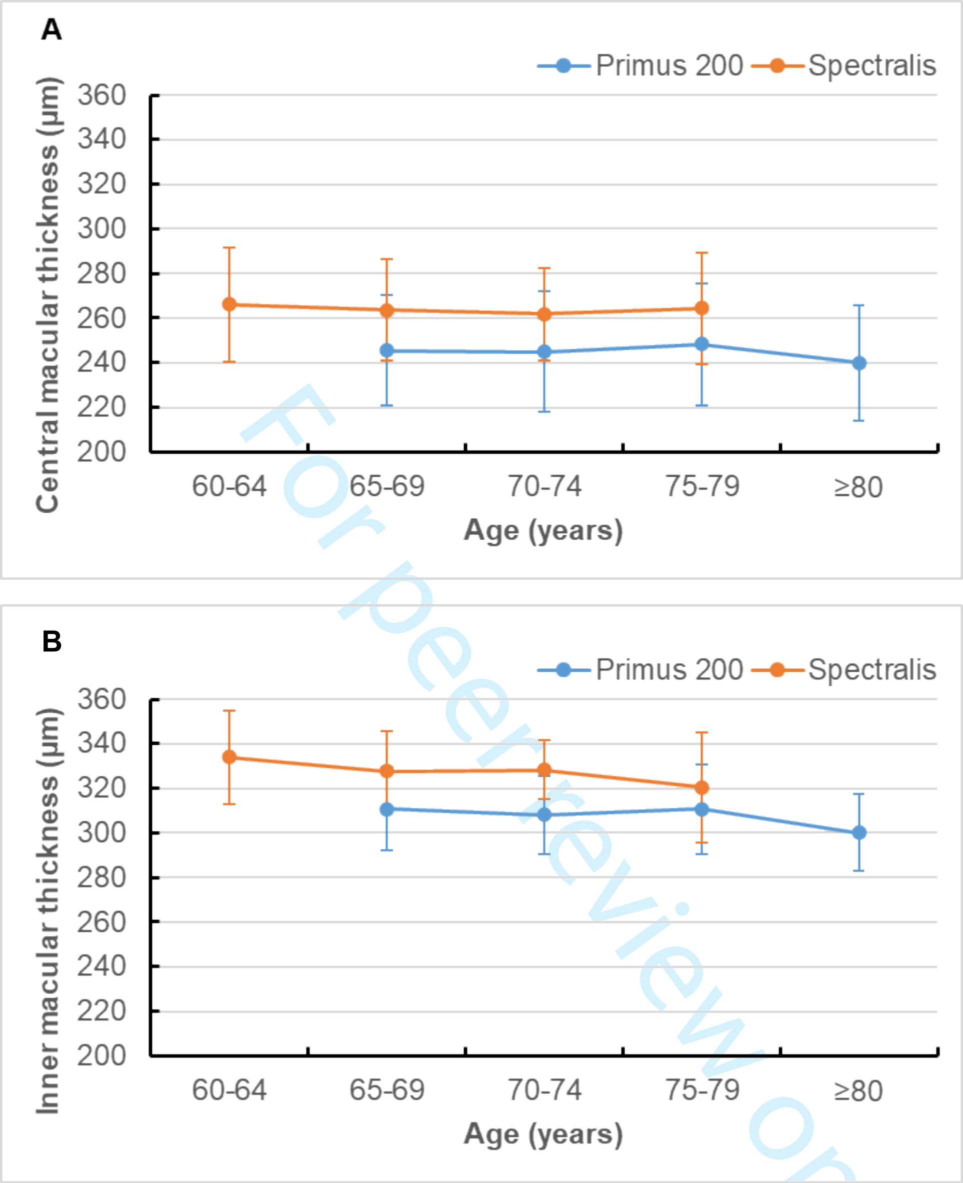

For peer review only

For peer review only - http://bmjopen.bmj.com/site/about/guidelines.xhtml

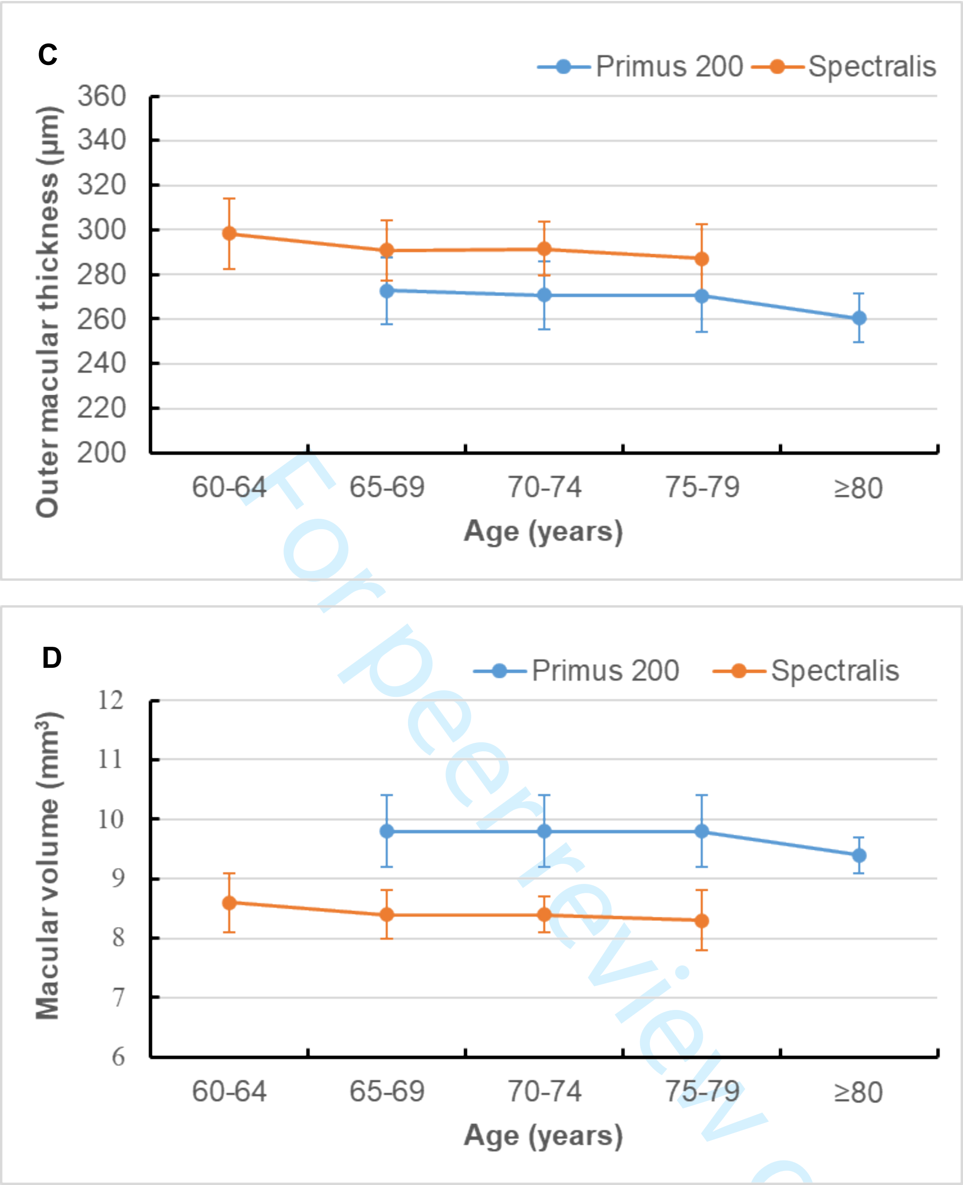

Figure 2. Mean (standard deviation) of macular thicknesses and volume assessed with Spectralis and Primus 200 OCT scanners by age groups. (A) Central macular thickness, (B) average inner macular thickness, (C) average outer macular thickness, and (D) macular volume.

For peer review only

For peer review only - http://bmjopen.bmj.com/site/about/guidelines.xhtml

STROBE Statement—Checklist of items that should be included in reports of ***cohort studies***

	Item No	Recommendation	Page No
**Title and abstract**	1	(*a*) Indicate the study's design with a commonly used term in the title or the abstract	1
		(*b*) Provide in the abstract an informative and balanced summary of what was done and what was found	2
**Introduction**			
Background/rationale	2	Explain the scientific background and rationale for the investigation being reported	3
Objectives	3	State specific objectives, including any prespecified hypotheses	3
**Methods**			
Study design	4	Present key elements of study design early in the paper	4
Setting	5	Describe the setting, locations, and relevant dates, including periods of recruitment, exposure, follow-up, and data collection	5-7
Participants	6	(*a*) Give the eligibility criteria, and the sources and methods of selection of participants. Describe methods of follow-up	4-5
		(*b*) For matched studies, give matching criteria and number of exposed and unexposed	
Variables	7	Clearly define all outcomes, exposures, predictors, potential confounders, and effect modifiers. Give diagnostic criteria, if applicable	5-7
Data sources/ measurement	8*	For each variable of interest, give sources of data and details of methods of assessment (measurement). Describe comparability of assessment methods if there is more than one group	6-7
Bias	9	Describe any efforts to address potential sources of bias	
Study size	10	Explain how the study size was arrived at	4
Quantitative variables	11	Explain how quantitative variables were handled in the analyses. If applicable, describe which groupings were chosen and why	6-7
Statistical methods	12	(*a*) Describe all statistical methods, including those used to control for confounding	7-8
		(*b*) Describe any methods used to examine subgroups and interactions	
		(*c*) Explain how missing data were addressed	
		(*d*) If applicable, explain how loss to follow-up was addressed	
		(*e*) Describe any sensitivity analyses	
**Results**			
Participants	13*	(a) Report numbers of individuals at each stage of study—eg numbers potentially eligible, examined for eligibility, confirmed eligible, included in the study, completing follow-up, and analysed	8
		(b) Give reasons for non-participation at each stage	25
		(c) Consider use of a flow diagram	25
Descriptive data	14*	(a) Give characteristics of study participants (eg demographic, clinical, social) and information on exposures and potential confounders	8
		(b) Indicate number of participants with missing data for each variable of interest	25
		(c) Summarise follow-up time (eg, average and total amount)	
Outcome data	15*	Report numbers of outcome events or summary measures over time	8-9

For peer review only

Main results	16	(*a*) Give unadjusted estimates and, if applicable, confounder-adjusted estimates and their precision (eg, 95% confidence interval). Make clear which confounders were adjusted for and why they were included<br>(*b*) Report category boundaries when continuous variables were categorized<br>(*c*) If relevant, consider translating estimates of relative risk into absolute risk for a meaningful time period	8-9
Other analyses	17	Report other analyses done—eg analyses of subgroups and interactions, and sensitivity analyses	
**Discussion**			
Key results	18	Summarise key results with reference to study objectives	10
Limitations	19	Discuss limitations of the study, taking into account sources of potential bias or imprecision. Discuss both direction and magnitude of any potential bias	12-13
Interpretation	20	Give a cautious overall interpretation of results considering objectives, limitations, multiplicity of analyses, results from similar studies, and other relevant evidence	13
Generalisability	21	Discuss the generalisability (external validity) of the study results	13
**Other information**			
Funding	22	Give the source of funding and the role of the funders for the present study and, if applicable, for the original study on which the present article is based	14

*Give information separately for exposed and unexposed groups.

Note: An Explanation and Elaboration article discusses each checklist item and gives methodological background and published examples of transparent reporting. The STROBE checklist is best used in conjunction with this article (freely available on the Web sites of PLoS Medicine at http://www.plosmedicine.org/, Annals of Internal Medicine at http://www.annals.org/, and Epidemiology at http://www.epidem.com/). Information on the STROBE Initiative is available at http://www.strobe-statement.org.

For peer review only

BMJ Open

Quantitative assessments of retinal macular structure among rural-dwelling older adults in China: a population-based, cross-sectional, optical coherence tomography study

Journal:	*BMJ Open*
Manuscript ID	bmjopen-2023-079006.R1
Article Type:	Original research
Date Submitted by the Author:	08-Jan-2024
Complete List of Authors:	zhang, qinghua; Shandong Provincial Hospital<br>Zhang, Cong; Shandong Provincial Hospital affiliated to Shandong University, Department of Neurology<br>Wang, Yongxiang; Shandong Provincial Hospital affiliated to Shandong University, Department of Neurology; Shandong Provincial Hospital Affiliated to Shandong First Medical University, Department of Neurology<br>Cong, Lin; Shandong Provincial Hospital affiliated to Shandong University, Department of Neurology; Shandong Provincial Hospital Affiliated to Shandong First Medical University, Department of Neurology<br>Liu, Keke; Shandong Provincial Hospital affiliated to Shandong University, Department of Neurology; Shandong Provincial Hospital Affiliated to Shandong First Medical University, Department of Neurology<br>Xu, Zhe; Shandong Provincial Hospital affiliated to Shandong University, Department of Neurology<br>Jiang, Chunyan; Shandong Provincial Hospital Affiliated to Shandong First Medical University, Department of Neurology<br>Zhou, Weiyan; Shandong Provincial Hospital affiliated to Shandong University, Department of Ophthalmology; Shandong Provincial Hospital Affiliated to Shandong First Medical University, Department of Ophthalmology<br>Zhang, Chunxiao; Shandong Provincial Hospital affiliated to Shandong University, Department of Ophthalmology; Shandong Provincial Hospital Affiliated to Shandong First Medical University, Department of Ophthalmology<br>Dong, Yi; Shandong Provincial Hospital affiliated to Shandong University, Department of Neurology<br>Feng, Jianli; Shandong Provincial ENT Hospital, Department of Neurology<br>Qiu, Chengxuan; Shandong Provincial Hospital affiliated to Shandong University, Department of Neurology; Stockholm University, Department of Neurobiology, Care Sciences and Society<br>Du, YiFeng; Shandong Provincial Hospital affiliated to Shandong University, Department of Neurology; Shandong Provincial Hospital Affiliated to Shandong First Medical University, Department of Neurology
**Primary Subject Heading**:	Epidemiology
Secondary Subject Heading:	Neurology
Keywords:	Epidemiology < TROPICAL MEDICINE, Aging, Neurology < INTERNAL

	MEDICINE, Diagnostic radiology < RADIOLOGY & IMAGING

SCHOLARONE™
Manuscripts

I, the Submitting Author has the right to grant and does grant on behalf of all authors of the Work (as defined in the below author licence), an exclusive licence and/or a non-exclusive licence for contributions from authors who are: i) UK Crown employees; ii) where BMJ has agreed a CC-BY licence shall apply, and/or iii) in accordance with the terms applicable for US Federal Government officers or employees acting as part of their official duties; on a worldwide, perpetual, irrevocable, royalty-free basis to BMJ Publishing Group Ltd ("BMJ") its licensees and where the relevant Journal is co-owned by BMJ to the co-owners of the Journal, to publish the Work in this journal and any other BMJ products and to exploit all rights, as set out in our licence.

The Submitting Author accepts and understands that any supply made under these terms is made by BMJ to the Submitting Author unless you are acting as an employee on behalf of your employer or a postgraduate student of an affiliated institution which is paying any applicable article publishing charge ("APC") for Open Access articles. Where the Submitting Author wishes to make the Work available on an Open Access basis (and intends to pay the relevant APC), the terms of reuse of such Open Access shall be governed by a Creative Commons licence – details of these licences and which Creative Commons licence will apply to this Work are set out in our licence referred to above.

Other than as permitted in any relevant BMJ Author's Self Archiving Policies, I confirm this Work has not been accepted for publication elsewhere, is not being considered for publication elsewhere and does not duplicate material already published. I confirm all authors consent to publication of this Work and authorise the granting of this licence.

For peer review only

Quantitative assessments of retinal macular structure among rural-dwelling older adults in China: a population-based, cross-sectional, optical coherence tomography study

Qinghua Zhang,[1,2] Cong Zhang,[1] Yongxiang Wang,[1,2] Lin Cong,[1,2] Keke Liu,[1,2] Zhe Xu,[1] Chunyan Jiang,[2] Weiyan Zhou,[3,4] Chunxiao Zhang,[3,4] Yi Dong,[1] Jianli Feng,[5] Chengxuan Qiu,[1,6*] Yifeng Du[1,2*]

1. Department of Neurology, Shandong Provincial Hospital affiliated to Shandong University, Jinan, Shandong, The People's Republic of China

2. Department of Neurology, Shandong Provincial Hospital affiliated to Shandong First Medical University, Jinan, Shandong, The People's Republic of China

3. Department of Ophthalmology, Shandong Provincial Hospital affiliated to Shandong University, Jinan, Shandong, The People's Republic of China

4. Department of Ophthalmology, Shandong Provincial Hospital affiliated to Shandong First Medical University, Jinan, Shandong, The People's Republic of China

5. Department of Neurology, Shandong Second Provincial General Hospital, Shandong Provincial ENT Hospital, Jinan, Shandong, The People's Republic of China

6. Aging Research Center and Center for Alzheimer Research, Department of Neurobiology, Care Sciences and Society, Karolinska Institutet-Stockholm University, Stockholm, Sweden

*Correspondence to:

Yifeng Du

Department of Neurology, Shandong Provincial Hospital, Cheeloo College of Medicine, Shandong University, No. 324 Jingwuweiqi Road, Jinan, Shandong 250021, China.

For peer review only

For peer review only - http://bmjopen.bmj.com/site/about/guidelines.xhtml

Email: duyifeng2013@163.com

Abstract

Objectives To quantitatively assess and compare retinal macular structures of rural-dwelling older adults in China using two different optical coherence tomography (OCT) scanners and to examine their associations with demographic, lifestyle, clinical, and ocular factors.

Design, setting and participants This population-based, cross-sectional study included 971 participants (age ≥60 years) derived from the Multimodal Interventions to Delay Dementia and Disability in Rural China (MIND-China) study. We collected data on demographics, lifestyle factors, clinical conditions (e.g., cardiovascular disease [CVD]), and ocular factors (e.g., visual acuity and spherical equivalent). We used two models of spectral-domain OCT to measure macular parameters in nine ETDRS subfields. Data were analysed using the multiple general linear models.

Results Spectralis OCT demonstrated higher macular thickness but a lower macular volume than Primus 200 OCT ($P<0.05$). Nasal quadrant of the inner and outer subfields was the thickest, followed by superior quadrant. Adjusting for multiple potential confounding variables, older age was significantly correlated with lower average inner and outer macular thicknesses and overall macular volume. Men had higher macular parameters than women. Presence of CVD was correlated with lower central macular thickness (β=-6.83; 95% CI: -13.08 to -0.58; P=0.032). Middle school or above was associated with higher average inner macular thickness (β=7.85; 95% CI: 1.14 to 14.55; P=0.022) and higher spherical equivalent was correlated with lower average inner macular thickness (β=-1.78; 95% CI: -3.50 to -0.07; P=0.042).

Conclusions Macular thickness and volume assessed by Spectralis and Primus 200 OCT scanners differ. Older age and female sex are associated with lower macular thickness and volume. Macular parameters are associated with education, CVD, and spherical equivalent.

For peer review only

For peer review only - http://bmjopen.bmj.com/site/about/guidelines.xhtml

Strengths and limitations of this study

- The strengths of our study include the population-based design that engages rural-dwelling older adults in China, the use of SD-OCT rather than TD-OCT scanners, and standardized assessment of systemic and ocular factors.
- The cross-sectional nature of our study does not allow us to make any causal inference for the observed associations between the examined factors and macular parameters.
- The observed cross-sectional associations are subject to selective survival bias that usually leads to underestimation of the true associations.
- Data on ocular factors were available only in a small sample, resulting in limited power to analyse associations of these factors with macular parameters.

For peer review only

For peer review only - http://bmjopen.bmj.com/site/about/guidelines.xhtml

INTRODUCTION

The retina develops from diencephalon and is an extension of the central nervous system (CNS) that exhibits typical properties of the brain[1]. In patients with neurodegenerative disorders such as Parkinson disease (PD) and Alzheimer disease (AD), macular changes may precede onset of symptoms in the CNS diseases[2-4]. Thus, changes of macular structure may be useful markers for early detection of CNS disorders.

Optical coherence tomography (OCT) is a key non-invasive imaging technique that enables clinicians to quantitatively measure macular thickness and volume in different subfields. The imaging speed (40,000 axial scans per second) and image resolution (axial resolution up to 5 μm) of OCT are much higher than that of MRI scans (resolution <100-500 μm), while the expenditure is much lower[5]. Therefore, OCT scan may provide a comprehensive understanding of the macular structure that can be implemented in studies of the general population settings.

In 2010, the population-based Handan Eye Study used the time-domain OCT to describe normal macular parameters in rural Chinese adults (n=2230, age 30-85 years)[6]. However, OCT technology has advanced remarkably from time-domain OCT to spectral-domain OCT (SD-OCT), which may provide more precise segmentation and higher resolution and measurement of retina[7]. Moreover, very few population-based studies have assessed the differences and agreements of macular measurements across various models of SD-OCT scanners. In addition, differences in macular parameters by demographic factors (e.g., age and sex) are well known, but ocular and vascular risk factors in association with macular measurements in older adults have not yet been well studied[8-11].

In this population-based study among rural-dwelling older adults in China, we sought to describe the methodology of quantitative assessments of the macular parameters assessed with SD-OCT scanners, to compare macular parameters measured using two different SD-OCT scanners, and further to examine the relations of macular parameters with demographic features, ocular factors, and vascular risk factors. We supposed that macular measurements assessed with different models of SD-OCT scanner differed and we further hypothesized that macular parameters (e.g., macular thicknesses and volume) in older adults were associated with demographic, lifestyle, clinical, and ocular factors.

For peer review only - http://bmjopen.bmj.com/site/about/guidelines.xhtml

METHODS

Study population

The study sample was derived from participants in the baseline examination of the Multimodal Interventions to Delay Dementia and Disability in Rural China (MIND-China) study, as previously described in detail[12]. Briefly, MIND-China targeted local rural residents who were aged ≥60 years by the end of 2017 and living in the 52 villages of Yanlou Town, Yanggu County, western Shandong Province, China. Baseline multidisciplinary assessments were conducted in March-September 2018, during which 5765 participants were examined[12].

In addition to testing the effectiveness of multimodal interventions to delay dementia and functional dependence in rural older adults, we intended to identify objective biomarkers for early detection of brain aging and cognitive impairment (e.g., mild cognitive impairment)[12]. Retinal measurements assessed with SD-OCT hold great potential for the early diagnosis, prognosis, and risk assessment of cognitive impairment[4]. The SD-OCT substudy was embedded within MIND-China and conducted at two local hospitals from March to June 2018 in Yanlou Town Hospital using Primus 200 OCT scanner (Carl Zeiss Meditec, Germany) and from June 2019 to November 2020 in Southwest Lu Hospital using Spectralis OCT scanner (Software Version 1.10.2.0; Heidelberg Engineering, Heidelberg, Germany). The cluster (village)-randomized sampling approach was used for selecting participants in the SD-OCT substudy. In total, 1083 subjects (76.7% of all eligible persons who undertook baseline examinations of MIND-China in March-June 2018) from 16 villages in Yanlou Town Hospital and 306 subjects (33.5% of all eligible persons who undertook the MRI substudy of MIND-China in June 2019-November 2020) from 20 villages in Southwest Lu Hospital underwent the SD-OCT scans. We excluded 383 persons scanned with Primus 200 and 35 persons scanned with Spectralis due to the following reasons: suboptimal image quality (e.g., signal strength less than six, segmentation and centration errors, and movement artifacts) (n=252 for Primus 200; n=21 for Spectralis), retinal disorders (e.g., epiretinal membrane, macular oedema, and macular hole) (n=125 for Primus 200; n=11 for Spectralis), and subjects with self-reported history of neurological disease (e.g., multiple sclerosis) (n=5 for Primus 200; n=3 for Spectralis), and subjects aged <65 years (n=1 for Primus 200). Finally, 700 subjects examined with the Primus 200 OCT, and 271 subjects assessed

For peer review only - http://bmjopen.bmj.com/site/about/guidelines.xhtml

with the Spectralis OCT were included in this analysis. **Figure 1** shows the flowchart of the study participants in the SD-OCT Substudy.

The protocol of the MIND-China study was reviewed and approved by the Ethics Committee at Shandong Provincial Hospital, Jinan, Shandong, China. Written informed consent was obtained from all participants, or in case of illiterate participants, from legally authorized representatives. MIND-China was registered in the Chinese Clinical Trial Registry (registration no.: ChiCTR1800017758).

Measurements and assessments of systemic factors

Data were collected via face-to-face interviews, clinical examination, laboratory test, and ocular examinations following the standard procedures[13]. Arterial blood pressure was measured using an electronic manometer (Omron HEM-7127J; Omron Corporation, Kyoto, Japan) after at least a 5-minute rest. After an overnight fast, peripheral blood samples were taken. Fasting serum glucose, total cholesterol, triglycerides, high-density lipoprotein cholesterol, low-density lipoprotein cholesterol were measured using an automatic Biochemical Analyzer (CS-600B, DIRUI Corporation, Changchun, China). Body mass index (BMI) was calculated as measured body weight (in kilograms) divided by body height (in meters) squared. We defined overweight as BMI 24-27.9 kg/m^2 and obesity as BMI ≥28 kg/m^2 according to recommendations for Chinese adults[14]. Smoking status was categorized into current, former, and never smoking. According to the frequency and quantity of alcohol consumption per week in the past 12 months, participants were divided into no or occasional drinkers and regular drinkers (at least once a week). Regular alcohol drinkers were further divided into light-to-moderate drinkers (<21 standard drinks/week for men and <14 standard drinks/week for women) and heavy drinkers (≥21 standard drinks/week for men, ≥14 standard drinks/week for women)[15]. Physical inactivity was defined as fewer than 150 minutes per week spent in moderate or vigorous physical activities[13]. Hypertension was defined as systolic blood pressure ≥140 mmHg, or diastolic blood pressure ≥90 mmHg, or current use of antihypertensive drugs[16]. Diabetes was defined as having a fasting plasma blood glucose level ≥7.0 mmol/L, or using hypoglycaemic drugs, or a self-reported history of diabetes[17]. Dyslipidaemia was defined as having a high-density lipoprotein level <1.0 mmol/L, or low-density lipoprotein level ≥4.1 mmol/L, or triglycerides level ≥2.3 mmol/L, or total

For peer review only - http://bmjopen.bmj.com/site/about/guidelines.xhtml

143 cholesterol ≥6.2 mmol/L, or using lipid-lowering drugs[13]. CVD was ascertained from in-person interviews, clinical and neurological examinations, and electrocardiogram examination, which included ischemic heart disease, heart failure, atrial fibrillation, and stroke[18].

146 **Measurements of ocular factors**

147 All participants underwent presenting visual acuity assessment and split-lamp examination. In Southwest Lu Hospital, participants underwent additional eye examinations, including best corrected vision acuity, IOP, spherical equivalent, axial length and fundus imaging by trained ophthalmologist. Presenting and best corrected vision acuity were examined using standard logarithmic visual acuity chart (Chinese edition, GB11533-2011) at a 5-meter distance. Visual impairment was defined as presenting distance visual acuity <4.5 in the better eye[19]. Intraocular pressure was examined using non-contact tonometer (TX-20, Canon, Japan). Spherical equivalent was examined using computerized auto refractor (HDR-7000 system, Huvitz, Korea). Axial length was measured using A-scan ultrasound biometry (Compact TouchV.4.00, Quantel Medical, France). The anterior segment of the eye was examined using a handheld portable slit-lamp microscope (BM900, Haag-Streit Diagnostics, Switzerland). Both disc-cantered and macular-centred fundus images were taken through undilated pupils using a hand-held direct ophthalmoscope (Suzhou Liuliu Vision Technology Co., Ltd., Suzhou, China).

160 **Imaging of optical coherence tomography**

161 The same procedure for SD-OCT examination was followed in the two hospitals. Following ocular examination, the SD-OCT scan was performed by experienced ophthalmic technicians. (1) The Primus 200 OCT has an acquisition rate of 12000 A-scans per second, with an axial resolution of 5 μm in tissue and a transversal resolution of <20 μm using a super luminescence diode with an 870 nm bandwidth[20]. We scanned only one eye and the right eye was the first choice. The structure of macular retinal layers was analysed following the macular thickness analysis protocol based on 512×32 macular cube scan in a dark room without pupil dilation, 6mm×6mm in scan area, centred at the fovea. (2) The Spectralis OCT has an acquisition rate of 40,000 A-scans per second, with an axial resolution of 5 μm in tissue and a transversal resolution of <6 μm using a super luminescence diode

For peer review only - http://bmjopen.bmj.com/site/about/guidelines.xhtml

with an 870 nm bandwidth. The built-in TruTrack active eye-tacking technology, which compensates for eye movement artifact, will improve the reproducibility of foveal centre selection during data acquisition[21]. Macular image was acquired using the perifoveal volumetric macular scan model (512 horizontal A scans per B scan, 49 horizontal B scans in 6 mm×6 mm area, no predetermined automatic real time) in a dark room without pupil dilation. The right eye was imaged first and then for the left eye.

Retinal thickness in the nine subfields defined by the Early Treatment Diabetic Retinopathy Study (ETDRS)[22] was acquired using the built-in software, as well as the general macular cube volume over the entire grid area. The standard retinal subfields are defined as central, inner superior, innerinferior, innernasal, innertemporal, outersuperior, outerinferior, outernasal, and outertemporal. The central foveal, inner, and outer subfields are bounded by the 1-mm, 3-mm, and 6-mm diameter circle, respectively. The whole retinal thickness was defined as the distance between internal limiting membrane (ILM) and retinal pigment epithelium boundary.

Evaluation of SD-OCT images

Quality control measures included image quality score, an internal limiting membrane (ILM) indicator, and motion indicators, which helped identify cases with segmentation errors, blinks, and eye motion artifacts[20 23 24]. ILM indicator was a measure of the minimum localized edge strength around the ILM boundary across the entire scan. It was useful for identifying segmentation errors and blink artifacts. The image quality of SD-OCT scans was assessed based on the range of the quality scores assigned by the respective instrument. For Primus 200 OCT, signal strength varies from 0-10, signal strength of 6 or more is considered adequate for image analysis[20]. For Spectralis OCT, the quality factor is a measure of signal-to-noise ratio expressed on a scale of 1 to 40 decibels. A quality reading of 25 decibels or more is considered adequate for image analysis[21].

Statistical analysis

IBM SPSS Statistics for Windows, Version 25.0 (IBM Corp. Released 2017. Armonk, NY, USA) was used for all statistical analysis. We presented mean (standard deviation, SD) for continuous variables and frequency (%) for categorical variables. We used one-way analysis of variance (ANOVA) or t test

For peer review only - http://bmjopen.bmj.com/site/about/guidelines.xhtml

to compare continuous variables, and chi-square test for categorical variables. Multiple general linear regression analyses were performed to examine the association of macular thickness and volumes (dependent variables) with various risk factors (independent variables). Adjustments were made first for age and sex in model 1, and additional adjustments were made for all other factors (i.e., education, smoking, alcohol consumption, CVD, and visual impairment) in model 2. The collinearity of the independent variables in model 2 was checked by calculating variance inflation factors (VIFs). Variables with high VIFs were excluded from the model. Data were missing in 3 participants (0.3%) for hypertension, and 7 (0.7%) for physical activity. Missing values in categorical variables were replaced with a dummy variable. For all analyses, a 2-tailed P value of less than 0.05 was considered statistically significant.

Patient and public involvement

None.

RESULTS

Characteristics of study participants

Characteristics of the study participants by two models of SD-OCT scanners were summarized in **Table 1**. Compared with participants who were scanned with Primus 200 OCT, those scanned with Spectralis OCT were slightly younger (mean age 68.1 vs. 70.4 years; P<0.001) and less likely to have cardiovascular disease (25.1% vs. 37.9%; P<0.001) but more likely to have visual impairment (7.7% vs. 4.0%; P=0.017). The two groups did not differ significantly in the distributions of sex, education, smoking, alcohol consumption, physical activity, BMI, hypertension, diabetes, and dyslipidaemia.

Comparation of macular parameters assessed with Spectralis and Primus 200

Compared with macular parameters of participants examined with Primus 200 OCT scanner, those scanned with Spectralis OCT scanner had significantly higher macular thickness in all ETDRS subfields but a lower macular volume (P<0.001) (**Table 2**). The association of macular parameters with type of scanner remained significant even after adjusting for age, sex, and education (P<0.001).

For peer review only - http://bmjopen.bmj.com/site/about/guidelines.xhtml

Correlates of macular parameters

As shown in **Figure 2**, measurements of inner and outer macular thickness and macular volume were significantly decreased with increasing age in participants measured with both scanners. There was no significant difference with age in central macular thickness of both samples of participants scanned with Primus 200 OCT and Spectralis OCT. In the multiple linear regression analysis, the association of age with macular thickness and volume remained significant even after further adjusting for multiple potential confounding variables, but the association of age with average inner macular thickness became statistically non-significant in the sample of participants examined with Primus 200 OCT (**Table 3**).

Female sex was significantly associated with lower central, average inner and outer macular thickness, and macular volume after adjusting for age in the sample of participants examined with both Spectralis OCT and Primus 200 OCT, and the association of female sex with average inner and outer macular thickness and macular volume in the sample of participants examined with Spectralis OCT and the association with central and average inner macular thickness in the sample of participants examined with Primus 200 OCT remained significant even after further adjusting for multiple potential confounding variables (**Table 3**).

In the sample of participants scanned with Spectralis OCT, after adjusting for sex and age, middle school or above was significantly associated with higher central and average inner macular thickness, and the association with average inner macular thickness remained significant after further adjusting for multiple potential confounding variables (**Table 3**). In addition, the presence of CVD was significantly associated with lower central macular thickness after adjusting for sex and age, but the association became statistically non-significant after further adjusting for multiple confounding factors. We also analysed the associations of ocular factors (i.e., IOP, axial length, and spherical equivalent) with macular parameters in the subsample of participants examined with Spectralis OCT. Higher spherical equivalent was significantly associated with lower average inner macular thickness after adjusting for sex and age, but the association became statistically non-significant after further adjusting for multiple confounding factors (**Table 3**). No significant associations of IOP and axial length with macular measurements were found (data not shown).

For peer review only - http://bmjopen.bmj.com/site/about/guidelines.xhtml

DISCUSSION

In this community-based SD-OCT scan study, we quantified macular thickness and volume among rural-dwelling Chinese older adults (age ≥60 years) using two models of SD-OCT scanners, compared macular parameters examined with Spectralis and Primus 200 OCT, and further explored demographics, lifestyle factors, clinical conditions, and ocular factors associated with macular thickness and volume.

Previous studies have shown that macular parameters measured with different SD-OCT scanners vary substantially[25]. We found that, compared with Primus 200 OCT, Spectralis OCT scanner yielded higher macular thickness but a lower macular volume, which was consistent with reports from the literature[25 26]. Different software algorithms were used in different OCT scanners, and the segmentation software of Spectralis OCT delineates the retinal pigment epithelium in a deeper layer than Primus 200 OCT [25]. This could be the main reason for higher macular thickness assessed with Spectralis OCT. In addition, the differences in macular parameters might be partially attributed to the fact that the sample of participants scanned with Spectralis OCT was younger than those scanned with Primus 200 OCT in our study.

We found that the macula was thinnest in the central subfields, followed by outer subfields and thickest in the inner subfields. Nasal quadrant of the inner and outer subfields was thickest, followed by the superior quadrant. These findings are in line with the distribution of neurons and synapse in the macular region of retina and consistent with reports from previous population-based studies [6 23 27-29]. The thicknesses of central, average inner and average outer macula of population scanned with Primus 200 OCT were slightly thinner than the reports from the Singapore Epidemiology of Eye Diseases Study of Chinese population (age ≥40 years)[27 28] and the UK Biobank study (age ≥40 years)[23]. However, macular thickness assessed with Spectralis OCT was thicker than that of the aforementioned reports. Differences in sociodemographic characteristics (e.g., age and sex) and OCT devices might partly contribute to the variation of macular thickness across studies[25].

In our study, older age was related to lower average inner and outer macular thickness and macular

For peer review only - http://bmjopen.bmj.com/site/about/guidelines.xhtml

volume. There was a trend towards decreasing in central macular thickness with increasing age, but the association was not significant. These results were consistent with the reports from previous studies[23 27 28 30] and were also in line with physiological characteristics of the retina[31 32]. Histological studies show that nerve fibres and ganglion cells with higher density in the peripheral part of the retina seem more vulnerable to loss with aging, while photoreceptors with higher density in the fovea are more resistant to age-related cell death[31 32].

Previous studies have consistently shown that women have thinner macular thickness in all subfields and smaller macular volume than men[6 23 27 29]. Our data showed similar sex differences in the examined macular parameters. Sex differences in macular thickness and volume may be attributed to hormonal changes at menopause, and this may explain why women were more likely than men to develop macular holes[29].

We also found that achieving middle school or above was associated with higher macular thickness in the sample of participants scanned with Spectralis OCT, but not in those scanned with Primus 200 OCT. The differences in demographics of both sample (e.g., mean age 68.1 for persons scanned with Spectralis vs. 70.4 years for persons scanned with Primus 200; $P<0.001$) and the different sensitivity of the two scanners might partly contribute to the discrepant results. The UK Biobank study showed that higher education attainment was associated with higher thickness of macular retinal nerve fibre layer, ganglion cell complex, and ganglion cell-inner plexiform layer, but the association between overall macular thickness and education attainment was not reported in this study[33]. Further large-scale population-based studies are needed to clarify the association of educational attainment with macular thickness.

Several population-based studies have investigated the associations of lifestyle factors and clinical conditions with macular measurements[6 9 23 27-29], and some studies found that alcohol consumption, smoking[23], high BMI[6 23], high fasting blood glucose[6] or diabetes[28], low levels of total cholesterol and triglyceride, and chronic kidney disease[28] were associated with lower macular measurements. Our data showed that CVD was associated with lower central macular thickness in population scanned with Spectralis OCT. However, we did not find associations of common lifestyle factors and other clinical conditions with macular parameters. The exact reasons for

For peer review only - http://bmjopen.bmj.com/site/about/guidelines.xhtml

306 the discrepant results across studies are not clear, but the differences in sociodemographic features of study population and some methodological issues across studies might play a part. Of note, our study participants were derived from a typical rural area in China, where older people had low socioeconomic status, the majority of them received no or very limited education, and they had distinct lifestyles from those of urban populations. Pathophysiologically, macular thickness could be affected by hypertension[23 27 28]. The retina undergoes various pathophysiologic changes in patients with hypertension, such as vasoconstriction, vascular sclerosis, and exudative changes. Vasoconstriction and vascular sclerosis may contribute to macular thinning, while exudative changes may induce abnormal macular thickening[9 34]. Therefore, it is important to evaluate ocular fundus changes before OCT examination. However, in our study only participants examined with Spectralis OCT in Southwest Lu Hospital underwent fundus imaging evaluation. In addition, macular thickness is affected by diabetic retinopathy and its severity[28 35]. Unfortunately, diabetic retinopathy was not evaluated in our study. Further studies with careful assessment of diabetic retinopathy may help determine the relationships of macular measurements with diabetes and complications. Regular physical activity may promote ocular health, but no previous studies have analysed the effect of physical activity on macular thickness. We found no association between physical inactivity and macular parameters among the rural-dwelling older adults.

Macular thickness and volume also varied with ocular factors. We found that lower spherical equivalent was associated with higher average inner macular thickness in the sample of participants scanned with Spectralis, which was consistent with reports from previous studies[23 36] and histologic findings of retinal thinning in myopic eyes[37 38]. However, as there was no autorefractor in Yanlou Town Hospital, spherical equivalent was not examined for participants scanned with the Primus 200 device. Further large-scale population-based studies are warranted to clarify the association of spherical equivalent with macular thickness.

The strengths of our study include the population-based design, the use of SD-OCT rather than TD–OCT scanners, and standardized assessment of wide range of systemic and ocular factors. Our study targeted older adults (age ≥60 years) who were living in the rural communities, whereas the Handan Eye Study was the only population-based OCT study in China that investigated macular structures

For peer review only - http://bmjopen.bmj.com/site/about/guidelines.xhtml

mainly among young and middle-aged urban residents (age ≥30 years, mean age 46.4 years)[6]. Furthermore, we examined a broader range of demographic, lifestyle, clinical, and ocular factors associated with macular parameters that were not reported in the previous study[6]. However, our study has limitations. First, ocular factors were acquired only in a small sample, thus, the study has limited power to analyse associations of these factors with macular parameters. Second, the cross-sectional nature of our study does not allow us to make any causal inference for the observed associations between factors studied and macular parameters, and the observed cross-sectional associations might be subject to selective survival bias that usually leads to underestimation of the true associations. Third, we were not able to control for the potential confounding effects of some ocular diseases (e.g., age-related macular degeneration and diabetic retinopathy) due to lack of assessments of these disorders, and residual confounding might also exist due to imperfect assessments of some confounding factors. Finally, our study samples were relatively younger, more educated, and more likely to smoke and have visual impairment compared with the total MIND-China sample, which should be kept in mind when generalizing our findings to other populations.

CONCLUSION

This population-based SD-OCT study of older adults who were free of ocular disorders quantified the macular thicknesses and volumes. We found that older age and female sex were associated with thinner macular thickness. Furthermore, Spectralis OCT produced higher macular thickness but a lower macular volume than Primus 200 OCT. These data may help interpret macular parameters in the diagnosis and management of retinal disorders in older adults. In addition, our study provides detailed description of baseline macular measurements in a subsample of the community-based MIND-China study, which is highly relevant for future follow-up studies that investigate the longitudinal relationships of macular parameters with measures of brain aging and functional phenotypes.

List of abbreviations

SD, standard deviation; SD-OCT, spectral-domain optical coherence tomography; BMI, body mass index; MIND-China, multimodal interventions to delay dementia and disability in rural China; CVD,

For peer review only - http://bmjopen.bmj.com/site/about/guidelines.xhtml

cardiovascular disease; IOP, intraocular pressure; ETDRS, early treatment diabetic retinopathy study; CNS, central nervous system; PD, Parkinson disease; AD, Alzheimer disease; MRI, magnetic resonance imaging; ILM, internal limiting membrane; SD, standard deviation; ANOVA, one-way analysis of variance.

Declarations

Ethics approval and consent to participate

The studies involving human participants were reviewed and approved by Ethics Committee at Shandong Provincial Hospital affiliated to Shandong University in Jinan, Shandong Province. All methods were performed in accordance with the relevant guidelines and regulations. All the patients/participants/legally authorized representatives of illiterate participants provided their written informed consent to participate in this study.

Consent for publication

Not applicable.

Data availability statement

The raw data supporting the conclusions of this article will be made available from the corresponding authors upon reasonable request and approval by the Steering Committee of MIND-China, without undue reservation.

Competing interests

The authors declare no financial or other conflicts of interest.

Acknowledgements

We would like to thank all participants of the MIND-China project as well as our research staff at Yanlou Town Hospital, Southwest Lu Hospital, and the Department of Neurology, Shandong

For peer review only

For peer review only - http://bmjopen.bmj.com/site/about/guidelines.xhtml

385 Provincial Hospital who were involved in the data collection and management.

386 **Funding**

387 This work was supported in part by grants from the National Key R&D Program of China (grant no.: 388 2017YFC1310100), the National Nature Science Foundation of China (grants no.: 82171175, 389 81861138008, and 81772448), the Academic Promotion Program of Shandong First Medical 390 University, the Taishan Scholar Program of Shandong Province, Shandong Province postdoctoral 391 innovation research program, and the Jinan Science and technology development plan (grant no. 392 201805076), China. C Qiu received grants from the Swedish Research Council (VR, grants no.: 2017- 393 05819 and 2020-01574), and the Swedish Foundation for International Cooperation in Research and 394 Higher Education (STINT, grant no.: CH2019-8320), and Karolinska Institutet, Stockholm, Sweden. 395 The funding agency had no role in the study design, data collection and analysis, the writing of this 396 manuscript, and in the decision to submit the work for publication.

397 **Contributors**

398 QZ, LC, YW, YDu, and CQ contributed to the study concept and design. QZ, CZ, ZX and CJ 399 contributed to the acquisition of data; QZ, YDong and JF contributed to the assessment of data. WZ 400 and CZ contributed to the assessment of OCT images; QZ and KL contributed to the data analysis. 401 QZ, WZ and CZ contributed to interpretation of the results; QZ contributed to the drafting of the 402 manuscript. YDu and CQ contributed to the study supervision. All authors contributed to the critical 403 revision of the manuscript for important intellectual content, article, and approved the submitted 404 version.

For peer review only - http://bmjopen.bmj.com/site/about/guidelines.xhtml

References

1. Cheung CY-L, Ikram MK, Chen C, et al. Imaging retina to study dementia and stroke. *Progress in retinal and eye research* 2017;**57**.
2. Ito Y, Sasaki M, Takahashi H, et al. Quantitative Assessment of the Retina Using OCT and Associations with Cognitive Function. *Ophthalmology* 2020;**127**(1):107-18.
3. Mutlu U, Colijn JM, Ikram MA, et al. Association of Retinal Neurodegeneration on Optical Coherence Tomography With Dementia: A Population-Based Study. *JAMA neurology* 2018;**75**(10):1256-63.
4. London A, Benhar I, Schwartz M. The retina as a window to the brain-from eye research to CNS disorders. *Nat Rev Neurol* 2013;**9**(1):44-53.
5. Cabrera DeBuc D, Gaca-Wysocka M, Grzybowski A, et al. Identification of Retinal Biomarkers in Alzheimer's Disease Using Optical Coherence Tomography: Recent Insights, Challenges, and Opportunities. *J Clin Med* 2019;**8**(7).
6. Duan XR, Liang YB, Friedman DS, et al. Normal macular thickness measurements using optical coherence tomography in healthy eyes of adult Chinese persons: the Handan Eye Study. *Ophthalmology* 2010;**117**(8):1585-94.
7. Seibold LK, Mandava N, Kahook MY. Comparison of retinal nerve fiber layer thickness in normal eyes using time-domain and spectral-domain optical coherence tomography. *Am J Ophthalmol* 2010;**150**(6):807-14.
8. Ooto S, Hangai M, Tomidokoro A, et al. Effects of age, sex, and axial length on the three-dimensional profile of normal macular layer structures. *Invest Ophthalmol Vis Sci* 2011;**52**(12):8769-79.
9. Kong M, Kwun Y, Sung J, et al. Association Between Systemic Hypertension and Macular Thickness Measured by Optical Coherence Tomography. *Investigative ophthalmology & visual science* 2015;**56**(4):2144-50.
10. Sasaki M, Kawashima M, Kawasaki R, et al. Association of serum lipids with macular thickness and volume in type 2 diabetes without diabetic macular edema. *Invest Ophthalmol Vis Sci* 2014;**55**(3):1749-53.
11. Sng CCA, Cheung CY, Man RE, et al. Influence of diabetes on macular thickness measured using optical coherence tomography: the Singapore Indian Eye Study. *Eye (Lond)* 2012;**26**(5):690-98.
12. Wang Y, Han X, Zhang X, et al. Health status and risk profiles for brain aging of rural-dwelling older adults: Data from the interdisciplinary baseline assessments in MIND-China. *Alzheimers Dement (N Y)* 2022;**8**(1):e12254.
13. Han X, Jiang Z, Li Y, et al. Sex disparities in cardiovascular health metrics among rural-dwelling older adults in China: a population-based study. *BMC Geriatr* 2021;**21**(1):158.
14. Zhou B-F. Predictive values of body mass index and waist circumference for risk factors of certain related diseases in Chinese adults--study on optimal cut-off points of body mass index and waist circumference in Chinese adults. *Biomed Environ Sci* 2002;**15**(1):83-96.
15. Järvenpää T, Rinne JO, Koskenvuo M, et al. Binge drinking in midlife and dementia risk. *Epidemiology (Cambridge, Mass)* 2005;**16**(6):766-71.
16. Jia P, Lee H, Chan J, et al. Long-Term Blood Pressure Variability Increases Risks of Dementia and Cognitive Decline: A Meta-Analysis of Longitudinal Studies. *Hypertension (Dallas, Tex : 1979)* 2021:HYPERTENSIONAHA12117788.
17. Liu X, Li Y, Li L, et al. Prevalence, awareness, treatment, control of type 2 diabetes mellitus and risk factors in Chinese rural population: the RuralDiab study. *Sci Rep* 2016;**6**:31426.
18. Cong L, Ren Y, Hou T, et al. Use of Cardiovascular Drugs for Primary and Secondary Prevention of

For peer review only

For peer review only - http://bmjopen.bmj.com/site/about/guidelines.xhtml

Cardiovascular Disease Among Rural-Dwelling Older Chinese Adults. *Frontiers in pharmacology* 2020;**11**:608136.

19. Vitale S, Cotch MF, Sperduto RD. Prevalence of visual impairment in the United States. *JAMA* 2006;**295**(18):2158-63.
20. Balasubramanian M, Bowd C, Vizzeri G, et al. Effect of image quality on tissue thickness measurements obtained with spectral domain-optical coherence tomography. *Opt Express* 2009;**17**(5):4019-36.
21. Ctori I, Huntjens B. Repeatability of Foveal Measurements Using Spectralis Optical Coherence Tomography Segmentation Software. *PLoS One* 2015;**10**(6):e0129005.
22. Relhan N, Flynn HW. The Early Treatment Diabetic Retinopathy Study historical review and relevance to today's management of diabetic macular edema. *Curr Opin Ophthalmol* 2017;**28**(3):205-12.
23. Patel PJ, Foster PJ, Grossi CM, et al. Spectral-Domain Optical Coherence Tomography Imaging in 67 321 Adults: Associations with Macular Thickness in the UK Biobank Study. *Ophthalmology* 2016;**123**(4):829-40.
24. Tewarie P, Balk L, Costello F, et al. The OSCAR-IB consensus criteria for retinal OCT quality assessment. *PLoS One* 2012;**7**(4):e34823.
25. Mylonas G, Ahlers C, Malamos P, et al. Comparison of retinal thickness measurements and segmentation performance of four different spectral and time domain OCT devices in neovascular age-related macular degeneration. *Br J Ophthalmol* 2009;**93**(11):1453-60.
26. Miller M, Zemon V, Nolan-Kenney R, et al. Optical coherence tomography of the retina in schizophrenia: Inter-device agreement and relations with perceptual function. *Schizophr Res* 2020;**219**:13-18.
27. Gupta P, Sidhartha E, Tham YC, et al. Determinants of macular thickness using spectral domain optical coherence tomography in healthy eyes: the Singapore Chinese Eye study. *Investigative ophthalmology & visual science* 2013;**54**(13):7968-76.
28. Wong KH, Tham Y-C, Nguyen DQ, et al. Racial differences and determinants of macular thickness profiles in multiethnic Asian population: the Singapore Epidemiology of Eye Diseases Study. *The British journal of ophthalmology* 2019;**103**(7):894-99.
29. Barrio-Barrio J, Ruiz-Canela M, Noval S, et al. Retinal Thickness Measured by Spectral-Domain Optical Coherence Tomography in Eyes Without Retinal Abnormalities: The Beaver Dam Eye Study. *Am J Ophthalmol* 2015;**160**(1):209-10.
30. Subhi Y, Forshaw T, Sørensen TL. Macular thickness and volume in the elderly: A systematic review. *Ageing research reviews* 2016;**29**:42-49.
31. Gao H, Hollyfield JG. Aging of the human retina. Differential loss of neurons and retinal pigment epithelial cells. *Invest Ophthalmol Vis Sci* 1992;**33**(1).
32. Panda-Jonas S, Jonas JB, Jakobczyk-Zmija M. Retinal photoreceptor density decreases with age. *Ophthalmology* 1995;**102**(12):1853-59.
33. Khawaja AP, Chua S, Hysi PG, et al. Comparison of Associations with Different Macular Inner Retinal Thickness Parameters in a Large Cohort: The UK Biobank. *Ophthalmology* 2020;**127**(1):62-71.
34. Ahn SJ, Woo SJ, Park KH. Retinal and choroidal changes with severe hypertension and their association with visual outcome. *Invest Ophthalmol Vis Sci* 2014;**55**(12):7775-85.
35. Dai W, Tham YC, Cheung N, et al. Macular thickness profile and diabetic retinopathy: the Singapore Epidemiology of Eye Diseases Study. *The British journal of ophthalmology* 2018;**102**(8):1072-76.
36. von Hanno T, Lade AC, Mathiesen EB, et al. Macular thickness in healthy eyes of adults (N = 4508) and relation to sex, age and refraction: the Tromsø Eye Study (2007-2008). *Acta ophthalmologica*

For peer review only

For peer review only - http://bmjopen.bmj.com/site/about/guidelines.xhtml

491 2017;**95**(3):262-69.
492 37. Chui TYP, Song H, Burns SA. Individual variations in human cone photoreceptor packing density: variations with refractive error. *Investigative ophthalmology & visual science* 2008;**49**(10):4679-87.
494 38. Grosvenor T, Scott R. Three-year changes in refraction and its components in youth-onset and early adult-onset myopia. *Optometry and vision science : official publication of the American Academy of Optometry* 1993;**70**(8):677-83.

497

For peer review only

For peer review only - http://bmjopen.bmj.com/site/about/guidelines.xhtml

Figure 1. Flowchart of study participants in the MIND-China SD-OCT substudy

Abbreviations: MIND-China, the Multimodal Interventions to Delay Dementia and Disability in Rural China; SD-OCT, spectral-domain optical coherence tomography.

Figure 2. Distributions of macular thicknesses and volume by age groups assessed with Spectralis and Primus 200 OCT scanners

Data are mean (standard deviation). (A) Central macular thickness, (B) average inner macular thickness, (C) average outer macular thickness, and (D) macular volume. Note: P was for the test of trend of macular parameters across age groups.

For peer review only

For peer review only - http://bmjopen.bmj.com/site/about/guidelines.xhtml

Table 1. Characteristics of the study participants in the analytical samples by OCT scanners

Characteristics	Primus 200 (n=700)	Spectralis (n=271)	P-value
**Demographic factors**			
Age, y, mean (SD)	70.4(4.2)	68.1(4.1)	<0.001
Female sex, n (%)	381(54.4)	153(56.5)	0.569
Education, n (%)			0.114
Illiterate	285(40.7)	91(33.6)	
Elementary school	297(42.4)	126(46.5)	
Middle school or above	118(16.9)	54(19.9)	
**Lifestyle factors**			
Smoking status, n (%)			0.110
Never	451(64.4)	192(70.8)	
Former	100(14.3)	27(10.0)	
Current	149(21.3)	52(19.2)	
Alcohol consumption, n (%)			0.657
No or occasional	495(70.7)	188(69.4)	
Light-to-moderate	169(24.1)	65(24.0)	
Heavy	36(5.1)	18(6.6)	
Physical inactivity, n (%)	447(63.9)	181(66.8)	0.391
**Clinical conditions**			
BMI (kg/m^2), n (%)			0.684
Normal (<24)	268(38.3)	112(41.3)	
Overweight (24-27.9)	291(41.6)	107(39.5)	
Obesity (≥28)	141(20.1)	52(19.2)	
Hypertension, n (%)	475(67.9)	186(68.8)	0.816
Diabetes, n (%)	94(13.4)	31(11.4)	0.406
Dyslipidaemia, n (%)	177(25.3)	60(22.1)	0.306
Cardiovascular disease, n (%)	265(37.9)	68(25.1)	<0.001
Visual impairment, n (%)	28(4.0)	21(7.7)	0.017

Abbreviations: SD, standard deviation; OCT, optical coherence tomography; BMI, body mass index.

In the analytical sample, data were missing in 3 participants (0.3%) for hypertension and 7 (0.7%) for physical activity. Missing values in categorical variables were replaced with a dummy variable.

P-value is for the test of differences between people scanned with Spectralis OCT and those scanned with Primus 200 OCT.

For peer review only

For peer review only - http://bmjopen.bmj.com/site/about/guidelines.xhtml

518 **Table 2.** Mean (SD) of macular thickness and volume in different macular subfields measured with
519 Spectralis and Primus 200 OCT scanners

**Macular parameters**	**Primus 200[a] (n=700)**	**Spectralis[a] (n=271)**	**β coefficient (95% CI)[b]**	**P-value**
Macular thickness(μm)				
Central macular subfield	245.6(26.0)	263.7(22.9)	17.98(14.43,21.53)	<0.001
Inner macular subfields				
Superior	310.9(23.3)	331.7(18.0)	19.55(16.43,22.67)	<0.001
Inferior	308.8(25.6)	327.6(20.8)	18.08(14.60,21.55)	<0.001
Nasal	314.8(20.2)	334.3(19.4)	18.86(16.07,21.66)	<0.001
Temporal	303.5(19.5)	319.9(18.5)	15.58(12.88,18.29)	<0.001
Average inner macular	309.5(18.6)	328.4(18.3)	18.02(15.42,20.61)	<0.001
Outer macular subfields				
Superior	277.6(20.9)	293.1(15.3)	13.79(10.99,16.59)	<0.001
Inferior	258.8(23.9)	283.9(15.7)	24.15(20.99,27.32)	<0.001
Nasal	289.2(19.3)	310.6(16.4)	20.13(17.48,22.79)	<0.001
Temporal	260.0(16.4)	281.0(14.6)	19.81(17.54,22.08)	<0.001
Average outer macular	271.4(15.3)	292.2(14.0)	19.47(17.35,21.60)	<0.001
Macular volume(mm^3)	9.8(0.6)	8.5(0.4)	-1.34(-1.42, -1.26)	<0.001

520 Abbreviation: OCT, optical coherence tomography; CI, confidence interval; SD, standard deviation.

521 [a]Data are mean (standard deviation).

522 [b]β coefficient (95% confidence interval) of macular parameters associated with scanner (Spectralis vs.
523 Primus 200), controlling for age, sex, and education.

For peer review only

For peer review only - http://bmjopen.bmj.com/site/about/guidelines.xhtml

Table 3. β coefficients (95% confidence intervals) of macular measurements associated with systemic and ocular factors from different models

Factors	**Central macular** (μm)				**Average inner macular** (μm)				**Average outer macular** (μm)				**Volume** (mm^3)
	Model 1	P-value	Model 2	P-value	Model 1	P-value	Model 2	P-value	Model 1	P-value	Model 2	P-value	Model 1
**Spectralis OCT(n=271)**													
Age (per 5 years)	-1.46 (-4.78, 1.85)	0.385	-1.01 (-4.36,2.33)	0.551	-3.62 (-6.17,-1.06)	0.006	-3.34 (-5.91,-0.77)	0.011	-3.43 (-5.42,-1.44)	0.001	-3.39 (-5.42,-1.36)	0.001	-0.10 (-0.15,-0.04)
Sex (female vs. male)	-10.45 (-15.86,-5.03)	<0.001	-5.32 (-13.67,3.03)	0.211	-11.63 (-15.80,-7.45)	<0.001	-10.20 (-16.61,-3.80)	0.002	-5.89 (-9.15,-2.63)	< 0.001	-5.13 (-10.19,-0.06)	0.047	-0.21 (-0.30,-0.11)
Education													
Illiterate	0.00 (ref.)		0.00 (ref.)		0.00 (ref.)		0.00 (ref.)		0.00 (ref.)		0.00 (ref.)		0.00 (ref.)
Elementary school	3.10 (-3.22,9.42)	0.335	2.96 (-3.44,9.35)	0.363	3.70 (-1.16, 8.55)	0.135	3.81 (-1.09,8.72)	0.127	2.12 (-1.70, 5.94)	0.275	2.35 (-1.52,6.23)	0.233	0.07 (-0.04, 0.18)
Middle school or above	8.86 (0.27,17.44)	0.043	8.24 (-0.50,16.98)	0.065	8.49 (1.90,15.09)	0.012	7.85 (1.14,14.55)	0.022	3.76 (-1.43, 8.94)	0.155	3.61 (-1.69,8.91)	0.181	0.14 (-0.01, 0.28)
Smoking													
Never	0.00 (ref.)		0.00 (ref.)		0.00 (ref.)		0.00 (ref.)		0.00 (ref.)		0.00 (ref.)		0.00 (ref.)
Former	-2.84 (-13.57,7.89）	0.603	-3.90 (-14.85,7.05)	0.484	-3.21 (-11.52, 5.10)	0.447	-4.32 (-12.72,4.08)	0.312	0.08 (-6.41, 6.58)	0.979	0.14 (-6.50,6.78)	0.967	-0.02 (-0.20, 0.17)
Current	4.93 (-3.78,13.65）	0.266	3.37 (-5.76,12.50)	0.468	-0.16 (-6.91, 6.58)	0.962	-1.31 (-8.32,5.69)	0.712	0.71 (-4.56, 5.98)	0.792	0.58 (-4.96,6.12)	0.837	0.02 (-0.13, 0.17)
Alcohol consumption													
No or occasional	0.00 (ref.)		0.00 (ref.)		0.00 (ref.)		0.00 (ref.)		0.00 (ref.)		0.00 (ref.)		0.00 (ref.)
Light-to-moderate	3.39 (-4.29,11.06）	0.386	1.74 (-6.17,9.65)	0.665	0.36 (-5.57, 6.29)	0.904	-0.69 (-6.77,5.38)	0.822	-1.73 (-6.35, 2.89)	0.462	-2.53 (-7.33,2.27)	0.300	-0.03 (-0.16, 0.10)
Heavy	3.94 (-7.83,15.71）	0.510	1.68 (-10.50,13.86)	0.786	0.64 (-8.45, 9.74)	0.889	0.81 (-8.54,10.16)	0.864	0.64 (-6.45, 7.72)	0.859	0.58 (-6.81,7.97)	0.878	0.02 (-0.18, 0.22)
CVD	-6.83 (-13.08,-0.58)	0.032	-6.05 (-12.41,0.31)	0.062	-3.09 (-7.94, 1.76)	0.211	-2.59 (-7.47,2.29)	0.296	-1.09 (-4.88, 2.70)	0.572	-1.01 (-4.87,2.85)	0.607	-0.05 (-0.16, 0.06)
Visual impairment	-1.50 (-11.61,8.62）	0.771	-0.03 (-10.26,10.20)	0.995	-4.79 (-12.58, 2.99)	0.226	-4.67 (-12.51,3.18)	0.243	-1.13 (-7.22, 4.96)	0.715	-1.07 (-7.28,5.14)	0.734	-0.05 (-0.23, 0.12)

For peer review only - http://bmjopen.bmj.com/site/about/guidelines.xhtml

Spherical equivalent	-0.27 (-2.51,1.97)	0.811	-0.09 (-2.34,2.16)	0.937	-1.78 (-3.50,-0.07)	0.042	-1.67 (-3.39,0.06)	0.058	-1.17 (-2.51, 0.17)	0.087	-1.15 (-2.51,0.21)	0.098	-0.04 (-0.07, 0.00)
**Primus 200 OCT(n=700)**													
Age (per 5 years)	-0.08 (-2.31,2.15)	0.941	0.10 (-2.16,2.36)	0.931	-1.47 (-3.07,0.12)	0.070	-1.36 (-2.98,0.26)	0.101	-2.34 (-3.67,-1.01)	0.001	-2.32 (-3.67,-0.97)	0.001	-0.07 (-0.12,-0.02)
Sex (female vs. male)	-13.31 (-17.07,-9.55)	＜0.001	-11.06 (-18.10,-4.02)	0.002	-8.72 (-11.41,-6.02)	＜0.001	-7.47 (-12.51,-2.43)	0.004	-4.32 (-6.57,-2.08)	＜0.001	-3.33 (-7.54,0.88)	0.121	-0.15 (-0.24,-0.07)
Education													
Illiterate	0.00 (ref.)		0.00 (ref.)		0.00 (ref.)		0.00 (ref.)		0.00 (ref.)		0.00 (ref.)		0.00 (ref.)
Elementary school	-1.90 (-6.39,2.59)	0.406	-2.02 (-6.55,2.50)	0.380	-0.35 (-3.57,2.88)	0.833	-0.58 (-3.82,2.66)	0.724	0.43 (-2.25, 3.12)	0.751	0.34 (-2.37,3.04)	0.807	0.00 (-0.10, 0.10)
Middle school or above	2.93 (-3.32,9.17)	0.358	2.94 (-3.34,9.22)	0.358	0.88 (-3.59,5.36)	0.699	0.62 (-3.87,5.12)	0.785	2.05 (-1.68, 5.78)	0.282	1.92 (-1.83,5.67)	0.315	0.09 (-0.05, 0.23)
Smoking													
Never	0.00 (ref.)		0.00 (ref.)		0.00 (ref.)		0.00 (ref.)		0.00 (ref.)		0.00 (ref.)		0.00 (ref.)
Former	1.50 (-6.16,9.15)	0.701	2.24 (-5.49,9.98)	0.570	2.90 (-2.58,8.37)	0.299	2.93 (-2.61,8.47)	0.300	1.58 (-2.99, 6.14)	0.497	1.83 (-2.79,6.46)	0.437	0.06 (-0.11, 0.24)
Current	3.31 (-3.79,10.40)	0.360	4.32 (-2.97,11.60)	0.245	-0.31 (-5.38,4.76)	0.905	-0.11 (-5.33,5.11)	0.966	-1.22 (-5.44, 3.01)	0.572	-1.14 (-5.50,3.21)	0.607	-0.08 (-0.24, 0.08)
Alcohol consumption													
No or occasional	0.00 (ref.)		0.00 (ref.)		0.00 (ref.)		0.00 (ref.)		0.00 (ref.)		0.00 (ref.)		0.00 (ref.)
Light-to-moderate	-2.34 (-7.52,2.85)	0.376	-2.71 (-8.00,2.57)	0.314	0.55 (-3.17,4.26)	0.773	0.64 (-3.15,4.43)	0.740	0.53 (-2.57, 3.63)	0.737	0.66 (-2.50,3.82)	0.681	0.03 (-0.09, 0.15)
Heavy	1.52 (-7.65,10.68)	0.745	0.59 (-8.74,9.93)	0.901	-1.41 (-7.97,5.15)	0.673	-1.12 (-7.81,5.56)	0.742	-0.28 (-5.75, 5.19)	0.920	0.03 (-5.55,5.61)	0.991	0.01 (-0.20,0.21)
CVD	-1.40 (-5.27,2.47)	0.477	-1.51 (-5.41,2.39)	0.448	-0.35 (-3.12,2.42)	0.802	-0.52 (-3.31,2.27)	0.715	-1.27 (-3.58, 1.03)	0.279	-1.39 (-3.72,0.94)	0.243	-0.03 (-0.12,0.06)
Visual impairment	-4.47 (-14.09,5.15)	0.362	-4.67 (-14.32,4.99)	0.343	-6.39 (-13.26, 0.48)	0.068	-6.19 (-13.10,0.73)	0.079	-0.54 (-6.28, 5.21)	0.854	-0.17 (-5.94,5.61)	0.955	-0.14 (-0.35, 0.08)

[a]β coefficients (95% confidence intervals) were adjusted for age and sex in model 1; and additionally adjusted for all other factors in the table in model 2.

For peer review only - http://bmjopen.bmj.com/site/about/guidelines.xhtml

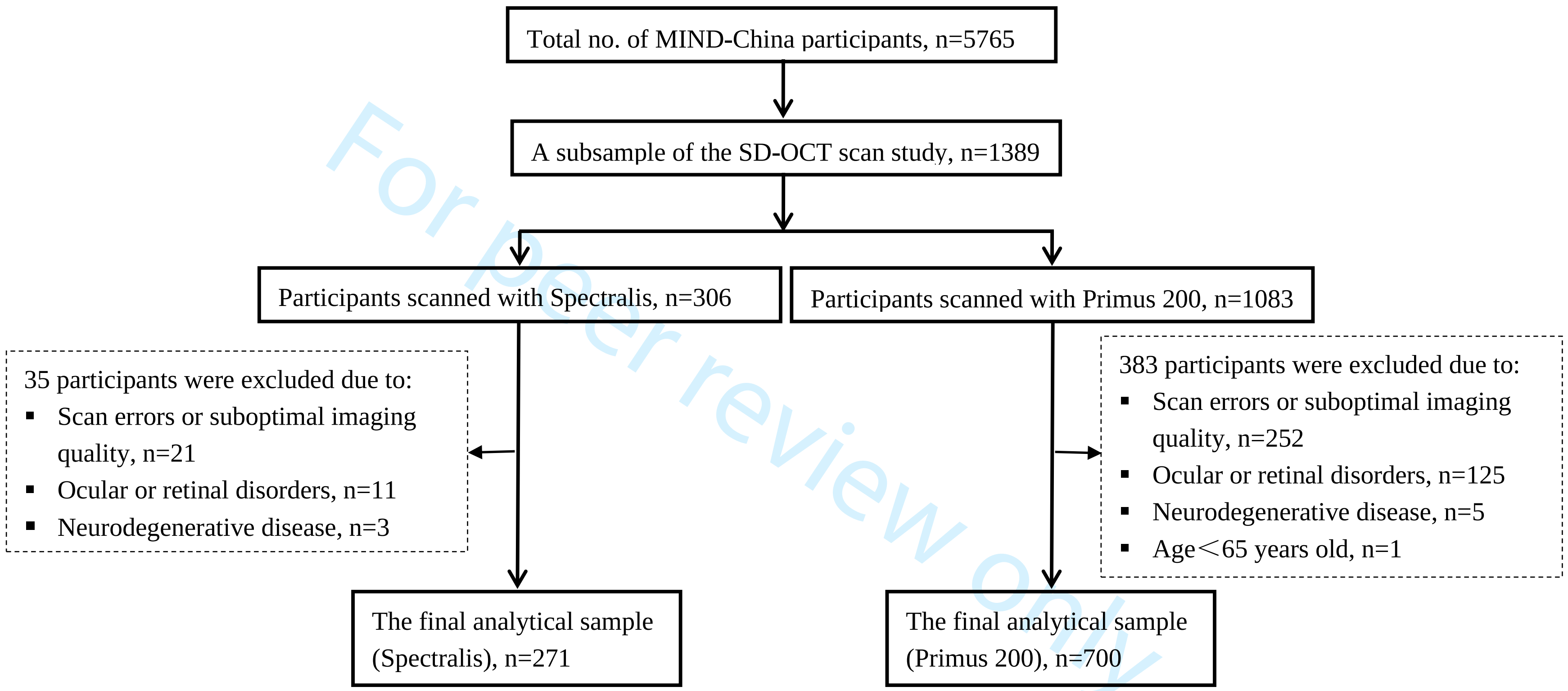

Figure 1. Flowchart of study participants in the MIND-China SD-OCT substudy

Abbreviations: MIND-China, multimodal interventions to delay dementia and disability in rural China; SD-OCT, spectral-domain optical coherence tomography.

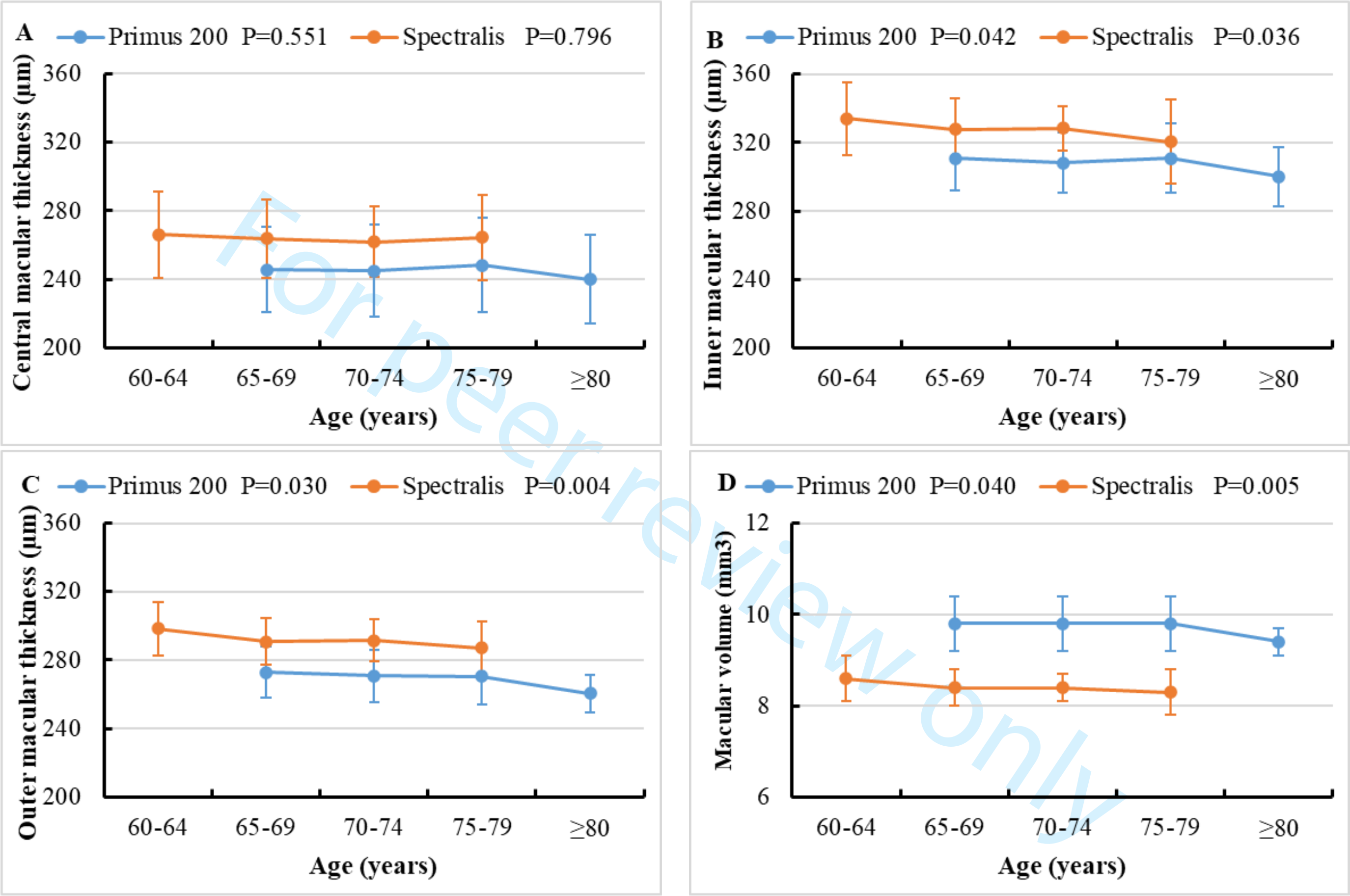

Figure 2. Mean (standard deviation) of macular thicknesses and volume assessed with Spectralis OCT (n=271) and Primus 200 OCT (n=700) by age groups.

(A) Central macular thickness, (B) average inner macular thickness, (C) average outer macular thickness, and (D) macular volume.

Note: P was for the test of trend of macular parameters across age groups.

For peer review only - http://bmjopen.bmj.com/site/about/guidelines.xhtml

STROBE 2007 (v4) Statement—Checklist of items that should be included in reports of *cross-sectional studies*

Section/Topic	Item #	Recommendation	Reported on page #
**Title and abstract**	1	(*a*) Indicate the study's design with a commonly used term in the title or the abstract	2
		(*b*) Provide in the abstract an informative and balanced summary of what was done and what was found	2
**Introduction**			
Background/rationale	2	Explain the scientific background and rationale for the investigation being reported	4
Objectives	3	State specific objectives, including any prespecified hypotheses	4
**Methods**			
Study design	4	Present key elements of study design early in the paper	5
Setting	5	Describe the setting, locations, and relevant dates, including periods of recruitment, exposure, follow-up, and data collection	5-8
Participants	6	(*a*) Give the eligibility criteria, and the sources and methods of selection of participants	5-6
Variables	7	Clearly define all outcomes, exposures, predictors, potential confounders, and effect modifiers. Give diagnostic criteria, if applicable	6-8
Data sources/ measurement	8*	For each variable of interest, give sources of data and details of methods of assessment (measurement). Describe comparability of assessment methods if there is more than one group	6-8
Bias	9	Describe any efforts to address potential sources of bias	
Study size	10	Explain how the study size was arrived at	5
Quantitative variables	11	Explain how quantitative variables were handled in the analyses. If applicable, describe which groupings were chosen and why	7-8
Statistical methods	12	(*a*) Describe all statistical methods, including those used to control for confounding	8-9
		(*b*) Describe any methods used to examine subgroups and interactions	
		(*c*) Explain how missing data were addressed	9
		(*d*) If applicable, describe analytical methods taking account of sampling strategy	
		(*e*) Describe any sensitivity analyses	
**Results**			

For peer review only

Participants	13*	(a) Report numbers of individuals at each stage of study—eg numbers potentially eligible, examined for eligibility, confirmed eligible, included in the study, completing follow-up, and analysed	5
		(b) Give reasons for non-participation at each stage	5
		(c) Consider use of a flow diagram	26
Descriptive data	14*	(a) Give characteristics of study participants (eg demographic, clinical, social) and information on exposures and potential confounders	9, 22
		(b) Indicate number of participants with missing data for each variable of interest	22
Outcome data	15*	Report numbers of outcome events or summary measures	
Main results	16	(*a*) Give unadjusted estimates and, if applicable, confounder-adjusted estimates and their precision (eg, 95% confidence interval). Make clear which confounders were adjusted for and why they were included	10-11, 23-25
		(*b*) Report category boundaries when continuous variables were categorized	6
		(*c*) If relevant, consider translating estimates of relative risk into absolute risk for a meaningful time period	
Other analyses	17	Report other analyses done—eg analyses of subgroups and interactions, and sensitivity analyses	
**Discussion**			
Key results	18	Summarise key results with reference to study objectives	11
Limitations	19	Discuss limitations of the study, taking into account sources of potential bias or imprecision. Discuss both direction and magnitude of any potential bias	14
Interpretation	20	Give a cautious overall interpretation of results considering objectives, limitations, multiplicity of analyses, results from similar studies, and other relevant evidence	14
Generalisability	21	Discuss the generalisability (external validity) of the study results	14
**Other information**			
Funding	22	Give the source of funding and the role of the funders for the present study and, if applicable, for the original study on which the present article is based	16

*Give information separately for cases and controls in case-control studies and, if applicable, for exposed and unexposed groups in cohort and cross-sectional studies.

Note: An Explanation and Elaboration article discusses each checklist item and gives methodological background and published examples of transparent reporting. The STROBE checklist is best used in conjunction with this article (freely available on the Web sites of PLoS Medicine at http://www.plosmedicine.org/, Annals of Internal Medicine at http://www.annals.org/, and Epidemiology at http://www.epidem.com/). Information on the STROBE Initiative is available at www.strobe-statement.org.

For peer review only
